# Nuclear Species-Diagnostic SNP Markers Mined from 454 Amplicon Sequencing Reveal Admixture Genomic Structure of Modern *Citrus* Varieties

**DOI:** 10.1371/journal.pone.0125628

**Published:** 2015-05-14

**Authors:** Franck Curk, Gema Ancillo, Frédérique Ollitrault, Xavier Perrier, Jean-Pierre Jacquemoud-Collet, Andres Garcia-Lor, Luis Navarro, Patrick Ollitrault

**Affiliations:** 1 Unité Mixte de Recherche Amélioration Génétique et Adaptation des Plantes (UMR Agap), Institut National de la Recherche Agronomique (Inra), Centre Inra de Corse, F-20230, San Giuliano, Corsica, France; 2 Centro de Protección Vegetal y Biotecnología, Instituto Valenciano de Investigaciones Agrarias (Ivia), 46113, Moncada, Valencia, Spain; 3 Unité Mixte de Recherche Amélioration Génétique et Adaptation des Plantes (UMR Agap), Centre de coopération Internationale en Recherche Agronomique pour le Développement (Cirad), TA A-108/02, 34398, Montpellier, Cedex 5, France; 4 Unité Mixte de Recherche Amélioration Génétique et Adaptation des Plantes (UMR Agap), Centre de coopération Internationale en Recherche Agronomique pour le Développement (Cirad), Station de Roujol, 97170, Petit-Bourg, Guadeloupe, France; USDA-ARS-SRRC, UNITED STATES

## Abstract

Most cultivated *Citrus* species originated from interspecific hybridisation between four ancestral taxa (*C*. *reticulata*, *C*. *maxima*, *C*. *medica*, and *C*. *micrantha*) with limited further interspecific recombination due to vegetative propagation. This evolution resulted in admixture genomes with frequent interspecific heterozygosity. Moreover, a major part of the phenotypic diversity of edible citrus results from the initial differentiation between these taxa. Deciphering the phylogenomic structure of citrus germplasm is therefore essential for an efficient utilization of citrus biodiversity in breeding schemes. The objective of this work was to develop a set of species-diagnostic single nucleotide polymorphism (SNP) markers for the four *Citrus* ancestral taxa covering the nine chromosomes, and to use these markers to infer the phylogenomic structure of secondary species and modern cultivars. Species-diagnostic SNPs were mined from 454 amplicon sequencing of 57 gene fragments from 26 genotypes of the four basic taxa. Of the 1,053 SNPs mined from 28,507 kb sequence, 273 were found to be highly diagnostic for a single basic taxon. Species-diagnostic SNP markers (105) were used to analyse the admixture structure of varieties and rootstocks. This revealed *C*. *maxima* introgressions in most of the old and in all recent selections of mandarins, and suggested that *C*. *reticulata* × *C*. *maxima* reticulation and introgression processes were important in edible mandarin domestication. The large range of phylogenomic constitutions between *C*. *reticulata* and *C*. *maxima* revealed in mandarins, tangelos, tangors, sweet oranges, sour oranges, grapefruits, and orangelos is favourable for genetic association studies based on phylogenomic structures of the germplasm. Inferred admixture structures were in agreement with previous hypotheses regarding the origin of several secondary species and also revealed the probable origin of several acid citrus varieties. The developed species-diagnostic SNP marker set will be useful for systematic estimation of admixture structure of citrus germplasm and for diverse genetic studies.

## Introduction

Citrus and its relatives are native in Southern to Eastern Asia, Malaysia, New Caledonia, and Australia [1]. The genus *Citrus* L. includes commercially important cultivars grown in tropical to temperate parts of the world over several thousands of years. Two major systems are widely used to classify *Citrus* species: the Swingle and Reece classification [1], which considers 16 species, and the Tanaka classification [2], which identifies 156 species. More recently, Mabberley [3] proposed a new classification of edible citrus recognising three species and four hybrid groups.

In this paper, we will refer to the Swingle and Reece [1] classification system widely used in the citrus scientist community. Despite the difficulties involved in establishing a consensual classification of edible citrus, molecular analyses provided decisive information for the comprehension of domestication and the relations between various cultivated species of *Citrus* [4–10]. These studies identified four ancestral taxa [*C*. *medica* L. (citron), *C*. *reticulata* Blanco (mandarin), *C*. *maxima* (Burm.) Merr. (pummelo), and *C*. *micrantha* Wester (papeda)] as the ancestors of all cultivated *Citrus*. The differentiation between these sexually compatible taxa may be explained by the foundation effect in different geographic zones and initial allopatric evolution. *Citrus maxima* originated in the Malay Archipelago and Indonesia, *C*. *medica* evolved in north-eastern India and the nearby region of Myanmar and China, *C*. *reticulata* diversification occurred over a region including Vietnam, southern China, and Japan [11,12] and *C*. *micrantha* seems to be originated from southern Philippian archipelago [1]. Moreover, diversity studies of morphological characteristics [13,14], primary metabolites [15], and secondary metabolites [16] proved that a major part of the phenotypic diversity of edible citrus resulted from differentiation between the basic taxa. Secondary species [*C*. *sinensis* (L.) Osb. (sweet orange), *C*. *aurantium* L. (sour orange), *C*. *paradisi* Macf. (grapefruit), *C*. *limon* (L.) Burm. (lemon), and *C*. *aurantifolia* (Christm.) Swing. (lime)] arose from hybridisations between the four basic taxa [5,7,8,10]. The partial apomixis of most of the secondary species has been an essential element limiting the number of further interspecific meiosis events. Therefore, most of the genomes of cultivated *Citrus* are mosaics of large chromosome fragments from the basic taxa in frequent interspecific heterozygosity. Another consequence of apomixis and horticultural vegetative propagation practices is that most citrus horticultural groups (sweet oranges, limes, lemons, grapefruits, clementines and satsumas) have minimal intragroup genetic diversity resulting from clonal variation/selection [17]. These horticultural groups are therefore particularly susceptible to emerging diseases. Moreover, conventional breeding of these varietal groups is hampered by the complex genetic structures that determine their specific phenotypes. Indeed, the highly heterozygous interspecific mosaic structure of their genome is broken by sexual recombination resulting in a very phenotypically heterogeneous progeny. However, useful natural phenotypic variability exists in the citrus gene pool, and traits are present for resistance to biotic and abiotic constraints [18]. The efficient utilization of this biodiversity in innovative breeding schemes will require prior insight into the phylogenetic origin and genomic structures of secondary species and modern cultivars. Recent whole genome sequencing projects [19,20] confirmed that *C*. *aurantium*, *C*. *sinensis*, and *C*. *clementina* (clementine) resulted from reticulation events between the *C*. *reticulata* and *C*. *maxima* gene pools and enabled to decipher the phylogenic origin of genomic fragments over the whole genome. However, the genomic structures of other secondary species and most modern varieties resulting from sexual crosses remain to be studied. For such objective it is essential to identify diagnostic molecular polymorphisms of the four citrus basic species throughout the genomes, and to develop molecular markers for routine phylogenetic genotyping of large germplasm collections. Moreover, diagnostic markers for ancestral taxa will aid the management of interspecific introgression in sexual breeding schemes and, more widely, will enable studies of sexual recombination at the diploid or polyploid levels and to analyse the mechanisms of 2n gamete formation for genotypes of interspecific origin.

Simple sequence repeat markers (SSRs) were widely developed in citrus during the last 15 years [21–25] SSRs are advantageous because they are highly polymorphic, codominant, generally locus-specific, and randomly dispersed throughout the genome [6,26,27]. However, Barkley *et al*. [28] showed that homoplasy may limit the usefulness of SSR markers in identifying the phylogenetic origin of DNA fragments in citrus. Garcia-Lor *et al*. [7] and Ollitrault *et al*. [29] showed that insertion/deletion polymorphisms (indels) and single nucleotide polymorphisms (SNPs) were more suitable to select efficient species diagnostic markers. Recent nuclear phylogenetic studies based on amplicon Sanger sequencing [10,30] revealed SNPs differentiating the four basic taxa. These SNPs were used for successful development of diagnostic SNP KASPar assays [9]. Several diagnostic SNPs for differentiation of *C*. *maxima* and *C*. *reticulata* were also identified from SNP markers developed from clementine Bac-end sequencing [8]. In addition, Wu *et al*. [20] revealed a huge number of diagnostic SNPs differentiating *C*. *maxima* and C. *reticulata* through analysis of whole genome sequencing (WGS) data from several mandarins and pummelos. However, the currently available validated species diagnostic markers [9] are still low in number, genome coverage is patchy, and specific markers are particularly scarce for *C*. *medica* and *C*. *micrantha*.

The objective of the present work was (i) to develop a set of species-diagnostic markers for the four *Citrus* ancestral taxa with coverage of the nine chromosomes of the citrus haploid genome, and (ii) to estimate the interspecific admixture genomic structure of the secondary cultivated species and several modern cultivars from 20^th^ century breeding programs. Specific diagnostic markers were mined from 454 amplicon sequencing of 57 gene fragments from 26 representative genotypes of the four basic taxa (eleven mandarins, nine pummelos, five citrons, and one papeda). Eighty-five SNP marker analyses based on competitive allele-specific PCR were developed. Effectiveness of marker development and transferability to related genera of the *Aurantioideae* subfamily is described in addition to the identification of ancestral alleles and validation of specific mutation occurrences in the four phylogenetic branches. Admixture analysis was performed using 73 markers successfully developed from the 454 SNP mining and 32 specific diagnostic SNP markers from previous research [8,9].

## Material and Methods

### Plant material

Leaves from 86 accessions of the *Citrus* genus and related genera were collected from pathogen-free plants from the IVIA Citrus Germplasm Bank (Valencia, Spain; accessions with IVIA identification numbers) and the Inra/Cirad citrus collection of San Giuliano (Corsica, France; accessions with SRA identification number, Table 1 and S1 Table). The Swingle and Reece [1] botanical classification was used for scientific names.

**Table 1:** Accession list.

Horticultural group	Latin name (Swingle and Reece, 1967)	Na
Mandarins	*Citrus reticulata* Blanco	14
	*Citrus tachibana* (Makino) Tanaka	3
Pummelos	*Citrus maxima* (Burm.) Merr	10
Citrons	*Citrus medica* L.	6
Papedas	*Citrus micrantha* Wester	2
Limes	*Citrus aurantiifolia* (Christm.) Swingle	4
Sour oranges	*Citrus aurantium* L.	3
Lemons	*Citrus limon* (L.) Burm. F.	6
Grapefruits	*Citrus paradisi* Macfad.	3
Sweet oranges	*Citrus sinensis* (L.) Osbeck	3
Combava	*Citrus hystrix* DC.	1
Amblycarpa	*Citrus reticulata* Blanco	1
Small citrus	*Citrus reticulata* Blanco	6
	*Citrus reticulata* Blanco *x Citrus paradisi Macf*.	3
	*Citrus reticulata* Blanco x *Citrus sinensis* (L.) Osb.	6
	*Citrus sinensis (L*.*) Osbeck x Citrus paradisi Macf*.	1
Other true citrus	*Citrus reticulata* hybrid (Calamondin)	1
	*Clymenia polyandra* (Tanaka)	1
	*Eremocitrus glauca* (Lindl.) Swingle	1
	*Fortunella crassifolia* Swingle	2
	*Microcitrus australis* (Planchon) Swingle	1
	*Poncirus trifoliata* (L.) Raf.	2
Near citrus	*Atalantia ceylanica* (Arn.) Oliv	1
	*Citropsis gilletiana* Swingle & M. Kellerm	1
Primitive citrus	*Severinia buxifolia* (Poir.) Tenore	1
*Triphasilinae*	*Triphasia trifolia* (Burm. F.) P.Wils.	1
*Clauseniae*	*Clausena excavata* Burm. f	1
	*Murraya koenigii* (L.) Spreng.	1

Na: number of accessions.

Twenty-six accessions representative of the four basic taxa (eleven mandarins [nine *C*. *reticulata* and two *C*. *tachibana*], nine pummelos [*C*. *maxima*], five citrons [*C*. *medica*], and one papeda [*C*. *micrantha*]) were used for SNP mining by 454 amplicon sequencing (Table 1 and S1 Table).

The study of admixture genomic structure of modern varieties using KASPar SNP markers was based on 70 *Citrus* accessions, including 24 of the 26 accessions noted above. For this study, the four ancestral taxa of the *Citrus* genus were represented by 33 accessions: seventeen mandarins, eight pummelos, six citrons, and two *C*. *micrantha* (S1 Table). Representatives of secondary citrus species included ten limes and lemons (four *C*. *aurantifolia*, six *C*. *limon*), three sour oranges (*C*. *aurantium*), three sweet oranges (*C*. *sinensis*), three grapefruits (*C*. *paradisi*), one ‘Combava’ *(C*. *hystrix*), and one *‘*Nasnaran’ mandarin (*C*. *amblycarpa*). Sixteen recent hybrid varieties from international breeding programs or supposed natural interspecific hybridisation were also used (four mandarin hybrids, eight tangors, including two clementines, according to recent demonstration of its origin [8,20], three tangelos, and one orangelo, [S1 Table]).

Transferability of the KASPar markers across the *Aurantioideae* subfamily was studied by the analysis of 14 accessions representative of the two tribes of the *Aurantioideae* (*Clausenae* and *Citreae*). In *Clausenae*, the subtribe *Clauseniae* was represented by two accessions (*Clausena excavata* Burm. f. and *Murraya koenigii* (L.) Spreng). Within the *Citreae*, two subtribes were represented: *Triphasilinae* (*Triphasia trifolia* (Burm. F.) P.Wils.; one accession), and *Citrinae* (eleven accessions representing nine genera including the *Citrus* genus). Analysis of the *Citrinae* was conducted according to the subdivision into three groups proposed by Swingle and Reece [1]: one accession of the “primitive citrus fruit” group (*Severinia buxifolia*), two accessions of two genera of the “near citrus fruit” group (*Atalantia ceylanica* (Arn.) Oliv and *Citropsis gilletiana* Swingle & M. Kellerm), and eight accessions of the “true citrus fruit trees” group that included six genera (two *Fortunella*, two *Poncirus*, one *Eremocitrus*, one *Microcitrus*, one *Clymenia*, and one *Citrus*, presumed of intergeneric origin) in addition to the *Citrus* species (S1 Table).

### DNA extraction

High molecular weight genomic DNA was extracted from leaf samples using the DNeasy Plant Mini Kit (Qiagen S.A.; Madrid, Spain) according to the manufacturer’s instructions.

### Target genomic fragment selection

The reference citrus whole genome sequence, released in Phytozome [31] by the International Citrus Genome Consortium, was used to select gene fragments. Annotated genes were acquired from: ftp://ftp.jgi-psf.org/pub/compgen/phytozome/v9.0/Cclementina/annotation/ at the Phytozome webpage (“Cclementina_182_gene.gff3” file).

Duplicated and overlapping genes were discarded. Then, from a specific annotation of the whole sequence for SSR (up to tetranucleotidic motifs and at least 11 bp sequences), all genes presenting microsatellite motifs were eliminated. For this study, 57 gene fragments covering the nine chromosomes were selected for SNP mining in genomic areas complementary to previously identified SNP marker sets [8,9].

### Amplicon library preparation

The 454 sequencing technique requires amplicon primers containing a directional GS FLX Titanium primer sequence (which includes a four base library “key” sequence) at the 5′ portion of the oligonucleotide in addition to the gene-specific sequence at the 3′ end. Multiplex Genotype Identifier (MID) sequences defined by Roche [32] (S2 Table) were added between the primer A (or B) and gene-specific sequences to allow for automated software identification of samples after pooling and sequencing [33].

For the 57 selected gene fragments (S3 Table), 57 primer pairs were designed according to the Access Array System for 454 Sequencing Platform User Guide [34] and loaded on the Fluidigm Access Array. The PCR products generated on the 48.48 Access Array IFC (Fluidigm 48.770 Digital PCR Workflow Quick Reference Card) were first analysed using an Agilent 2100 Bioanalyzer (Agilent DNA 1000 Kit Guide) to check the quality of the PCR products. Next, the PCR products were pooled in equal volume to create one PCR product library. The PCR product library was purified using AMPure beads. After purification, the PCR product library was quantified before proceeding to emulsion PCR. The PCR product library was quantified using the Quant-iT PicoGreen fluorimetry system (Quant-iT PicoGreen User Guide).

### Sequencing and sequence data analysis for SNP calling

Raw reads obtained from 454 pyrosequencing were pre-processed by removing low quality reads and adapter/primer sequences using PRINSEQ [35]. We considered as low quality reads the short reads (<150 Bases) with primers dimmers. The other reads, were automatically identified and sorted by MID and specific gene primers using SFF Tool commands of Newbler software [36].

For each sequenced gene of each variety, 454 pyrosequencing reads were aligned independently using SeqMan NGen software version 7.0 [37] with the following assembling parameters: Match size: 12; minimum Match Percentage: 80; Minimum Sequence length: 150. Consensus genomic sequences were generated from alignments.

Raw data are available in EMBL-EBI (European Nucleotide Archive), under the study accessions number: PRJEB8550

### KASPar genotyping

SNP genotyping was performed using KASPar technology (KBioscience; http://www.kbioscience.co.uk/). The KASPar Genotyping System is a competitive, allele-specific dual Förster resonance energy transfer (FRET)-based assay for SNP genotyping. Primers were designed by LGC Genomics based on the SNP locus-flanking sequence (approximately 50 nucleotides either side of the SNP). Detailed information for all SNP markers can be found in S4 Table. Additional details about this genotyping method can be found in Cuppen [38].

The fluorescence signals of PCR products were measured with Fluostar Omega (BMG) and genotype calling was made with KlusterCaller software (LGC Genomics).

### Genetic analysis of the SNP data

SNP numbers and locations were identified from sequence data using SniPlay online software [39,40].

Unbiased expected heterozygosity (He), observed heterozygosity (Ho), and F Stat parameters (F_w_ [41] and F_ST_) were calculated using Weir & Cockerham [42] estimator from the GENETIX v. 4.03 software [43].

The search for SNPs diagnostic for each taxon was based on G_ST_ parameter [44] estimations for the concerned taxa considering two subpopulations: (1) the concerned taxon (Ti), and (2) a theoretical population of the three other basic taxa (T-i). Analysis was performed from the estimated allele frequency of each taxon considering the same population size for each taxon to estimate the frequency of the two subpopulations (Ti and T-i) and the whole population (Tot) frequency. G_ST_ estimations were computed using Excel software:

$${\text{G}}_{\text{ST Taxoni}}=\frac{{\text{He}}_{\text{Tot}}-\frac{{\text{He}}_{\text{Ti}}+{\text{He}}_{\text{T}-\text{i}}}{2}}{{\text{He}}_{\text{Tot}}}$$

where He is the expected proportion of heterozygous loci per individual (He = 1 − ∑ pi^2^, where pi is the frequency of a given allele in the considered population or subpopulation). Values of G_ST_ range from 0 to 1. Low values indicate that little variation is proportioned among subpopulations, while high values denote that a large amount of variation is found among subpopulations. In this study, G_ST Taxoni_ = 1 indicated that the taxon i was totally differentiated from the three other basic taxa and probably fixed for a mutant allele that most likely occurred in the taxon i after its separation. SNPs presenting a GST value >0.9 where considered as diagnostic SNP for the corresponding taxon. For G_ST_ values <0.5, a SNP was considered as specific for a taxon when it was polymorphic within the taxon but fixed for the same allele in the other three taxa. The same criteria were used to select 32 additional diagnostic markers from SNP markers previously developed by our group [8,9].

Factorial Analyses (FA) from fragment sequences were performed using DARwin software [45].

Principal Component Analysis (PCA) was performed using XLSTAT software from allelic frequencies.

Genotypic genetic relationships were studied by Neighbour-joining analysis (NJA) based on the SNP data using DARwin software [45] with the simple matching dissimilarity index. This simple matching dissimilarity index was also used to infer the intra- and inter-taxa average differentiation.

Population structure was inferred using the Structure program version 2.3.4 [46], which implemented a model-based clustering method using genotype data [47,48]. No *a priori* population structure was defined. The linkage model option was used, with allele frequencies correlated and computed probability of the data for K estimating. Analyses were made with K value (number of subpopulations) varying from 1–10. The statistics used to select the correct K value was ΔK as proposed in Evanno *et al*. [49]. Ten runs of Structure were performed with 50,000 steps of burning followed by 50,000 Monte Carlo Markov Chain (MCMC) repetitions. For the better K value, the ten independent Structure run cluster outputs were permuted and aligned, and average frequency and standard error of the contribution of each basic population was estimated. The relative genetic distance between successive markers was directly obtained from the reference clementine genetic map [50] or inferred from the physical position of the markers using the curves relating genetic map position to physical location [20].

## Results

### 454 SNP mining and genotype calling

**Fig 1:**
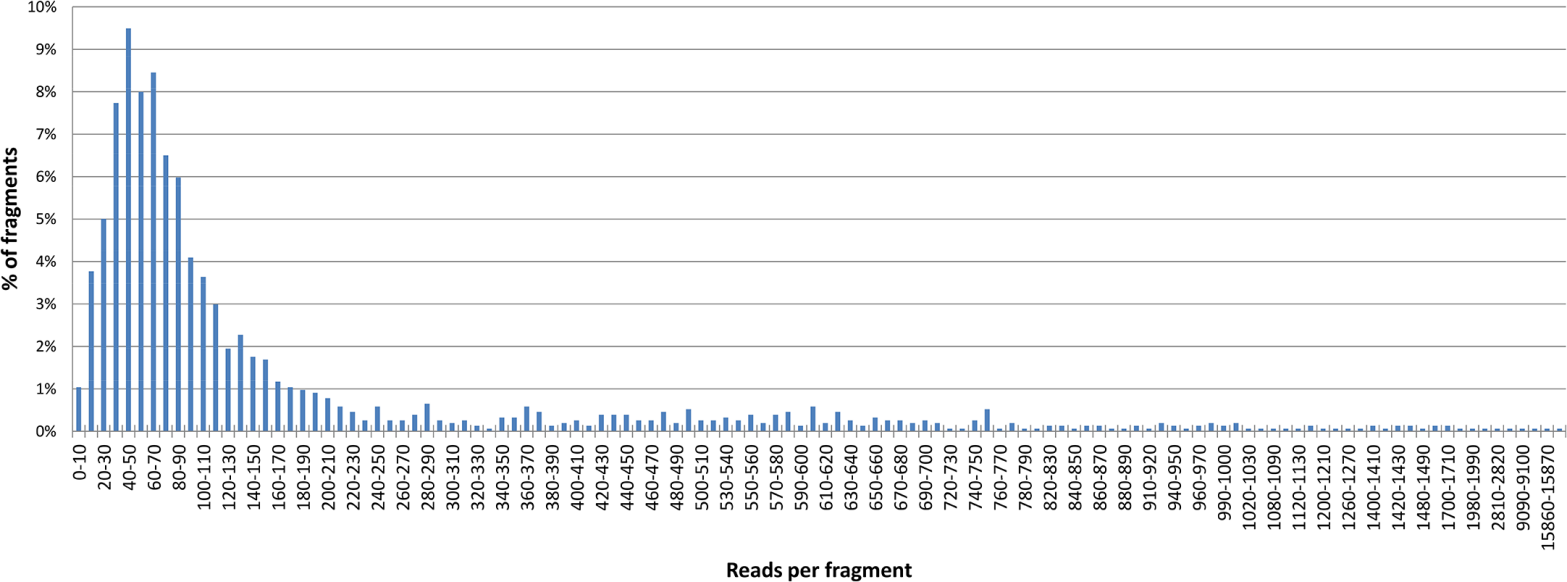
Distribution of read numbers per fragment.

Fluidigm amplification followed by 454 sequencing produced 295,169 useful reads. The reads were classified according to their MID, and then Titanium sequences and MID sequences were removed using 454 software tools. All reads were attributed to one of the 1,482 (57 × 26) amplicons according to the fragment gene sequence. Forty-six gene fragment/variety amplicons did not have a corresponding read (3.1%), and eight had insufficient read numbers for genotype calling. The average of reads per amplicon was 205.55 (for 1,436 amplicons with reads). However, the distribution of the number of reads per amplicon was highly heterogeneous (Fig 1), and 414 amplicons (27.94% of total gene fragments/varieties) had fewer than 50 reads.

For *C*. *micrantha* (represented by a single accession), gene fragments with missing data were Sanger sequenced to complete the data set.

A total of 1,053 SNPs were identified over 28,507 bp readable sequence for 57 gene fragments (Table 2). SNP genetic diversity parameters (Table 3 and S5 Table) were calculated for each SNP position, with a F_ST_ value of 0.445±0.020. The SNP/Kb rate varied between gene fragments (S6 Table; range: 11.19 [fragment C2P25] to 93.31 [fragment C7P8]) and chromosomes (Table 2; range: 30.48–50.10 SNP/kb in chromosomes 1 and 7, respectively). No significant differences were found between chromosomes for Ho, He, and Fw.

**Table 2:** Average SNP diversity by chromosome.

	Sequence size (bp)	Number of fragments per chromosome	SNP / chromosome	SNP / kb	Ho	He	Fw
Chr 1	3,084	6	94	30.48	0.049 ± 0.021	0.229 ± 0.034	0.582 ± 0.093
Chr 2	3,516	7	111	31.57	0.088 ± 0.026	0.231 ± 0.030	0.455 ± 0.080
Chr 3	2,543	5	79	31.06	0.055 ± 0.020	0.249 ± 0.040	0.594 ± 0.093
Chr 4	3,839	8	152	39.59	0.044 ± 0.013	0.232 ± 0.028	0.588 ± 0.072
Chr 5	2,475	5	71	28.69	0.059 ± 0.017	0.215 ± 0.037	0.501 ± 0.107
Chr 6	3,408	7	105	30.81	0.052 ± 0.015	0.221 ± 0.031	0.577 ± 0.087
Chr 7	2,475	5	124	50.10	0.073 ± 0.024	0.211 ± 0.027	0.420 ± 0.082
Chr 8	4,142	8	187	45.15	0.071 ± 0.015	0.218 ± 0.022	0.546 ± 0.062
Chr 9	3,025	6	130	42.98	0.074 ± 0.023	0.265 ± 0.031	0.623 ± 0.066
Total	28,507	57	1,053	36.94	0.064 ± 0.009	0.230 ± 0.010	0.544 ± 0.027

Ho: observed heterozygosity; He: Nei diversity index; Fw: Wright fixation index.

**Table 3:** SNP diversity of horticultural groups.

	SNPs	SNPs / kb	Ho	He	Fw
Pummelos (Na = 9)	297	10.42	0.056 ± 0.008	0.072 ± 0.004	0.171 ± 0.052
Citrons (Na = 5)	132	4.63	0.038 ± 0.018	0.043 ± 0.003	0.146 ± 0.025
*C*. *micrantha* (Na = 1)	71	2.49	0.068	0.034 ± 0.008	-
Mandarins (Na = 11)	445	15.61	0.082 ± 0.011	0.102 ± 0.006	0.128 ± 0.012

Na: Number of accessions; Ho: observed heterozygosity; He: Nei diversity index; Fw: Wright fixation index.

**Fig 2:**
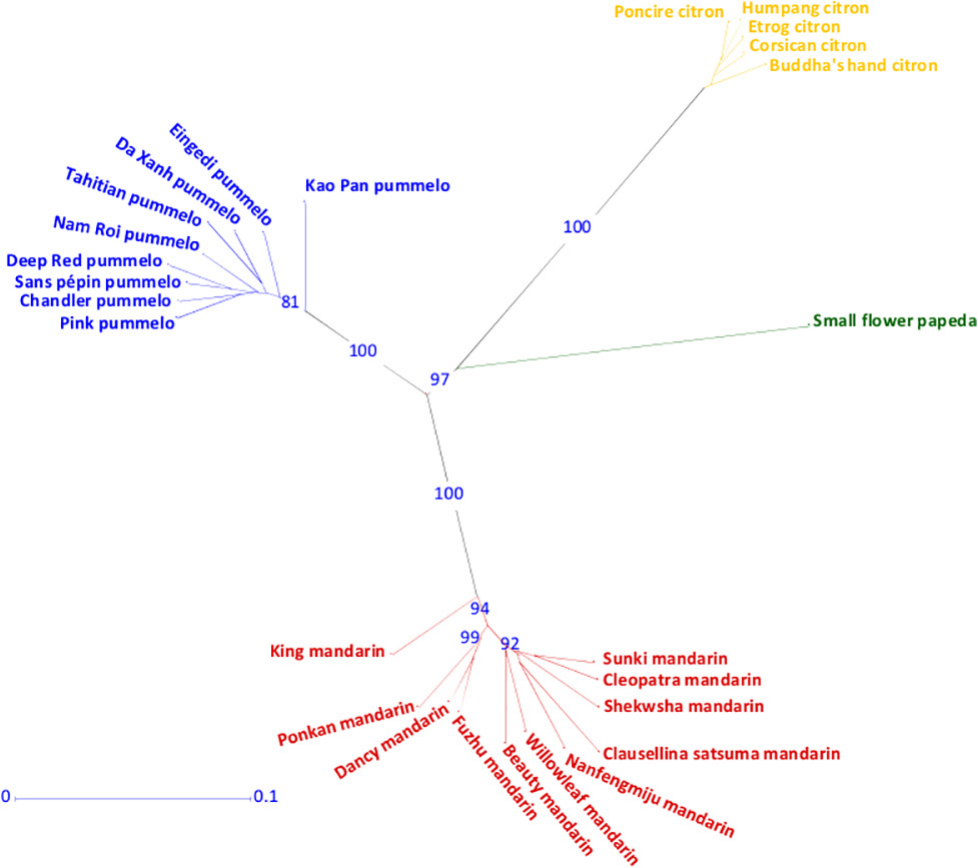
Neighbour-joining analysis (NJA) of representative of the four basic taxa based on the 1,053 SNPs found in all gene fragments.

The values of the fixation index over the whole population (Fw; 0.54) and average F_ST_ value considering the four horticultural groups as four subpopulation (0.45) suggest an important structuration of the analysed varietal sample, as confirmed by the NJA (Fig 2).

Intra-horticultural group Fw values ranged from 0.128 in mandarins to 0.171 in pummelos. Mandarin displayed the higher intragroup diversity (15.61 SNP/Kb and He = 0.102 ± 0.006). Citron displayed low heterozygosity (Ho = 0.04 ± 0.02) and polymorphism (He = 0.04 ± 0.003) compared with mandarins and pummelos. Only one representative of *C*. *micrantha* was available, and its observed heterozygosity value (0.07) lay between the pummelo and mandarin values (Table 3).

The analysis of the average number of SNPs/kb between two varieties within and between the four supposed basic taxa revealed values of 1.62–3.49 SNPs/kb within groups and 9.96–13.19 SNPs/kb at the inter-group level (Table 4).

**Table 4:** Intra- and inter-horticultural group dissimilarities (Average SNP/kb between two varieties).

	Mandarins	Pummelos	Citrons
Mandarins	3.49*		
Pummelos	9.96	2.61*	
Citrons	13.19	11.54	1.62*
*C*. *micrantha*	11.82	9.96	12.49

*Average number of SNP/kb at the intra-horticultural group level.

### Determination of species-diagnostic SNPs

An initial analysis of taxa differentiation at each SNP position was performed directly from genotype calls from the representative of each of the four basic taxa. Diagnostic SNP assignment was based on the G_ST_ parameter as described in the Materials and Methods. The distribution of the highest G_ST_ value of each SNP for the four basic taxa (S1 Fig and S5 Table) showed that 47% of the highest SNP G_ST_ values were >0.5. Moreover, *C*. *medica* (112 diagnostic SNPs) and *C*. *micrantha* (91) displayed many more diagnostic SNPs (G_ST_ >0.9) than *C*. *reticulata* (42) or *C*. *maxima* (26).

Among the SNPs with higher GST values <0.5, 501 were polymorphic within one taxon but fixed for the same allele in the other three taxa. For example, the 55 *C*. *micrantha* SNPs with GST values of 0.3–0.4 corresponded with heterozygous SNPs in *C*. *micrantha* fixed for a same allele in mandarin, pummelo, and citron. No specific allele was observed for 92 SNPs with the highest taxon G_ST_ value ≤0.5 (similar patterns of diversity in at least two basic taxa, denoted “N.D.” in S1 Fig).

**Fig 3:**
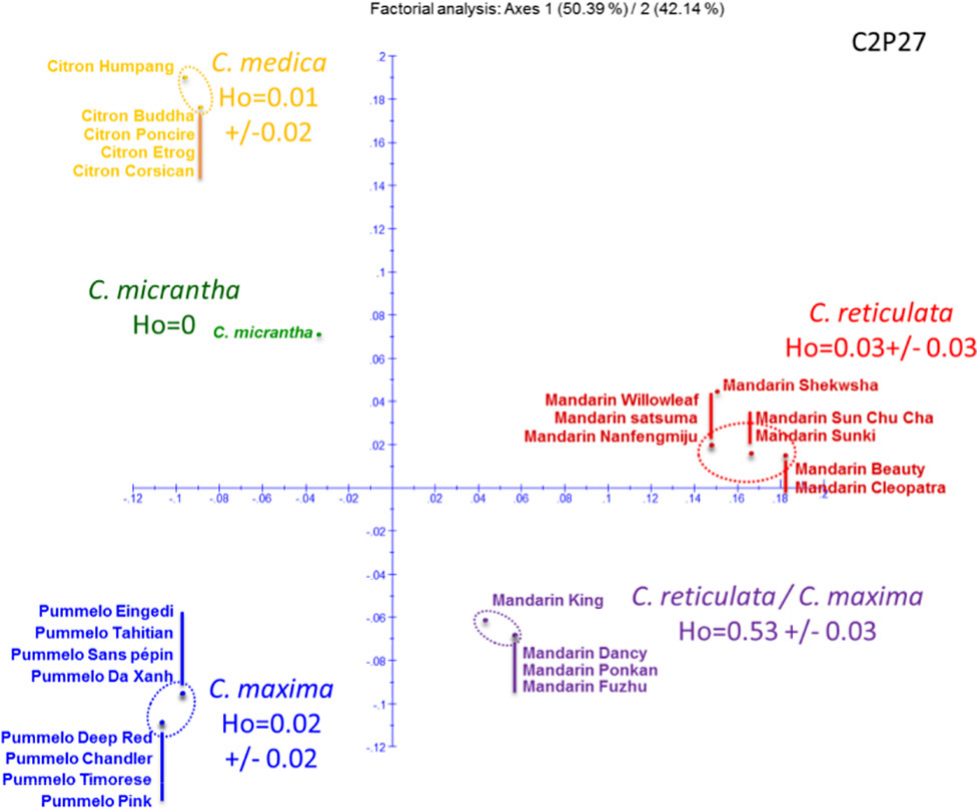
Factorial analysis and accession heterozygosity reveal *C*. *reticulata*/*C*. *maxima* heterozygosity of four mandarins for the C2P27 gene fragment.

Previous WGS [20] and amplicon sequencing [10,51] studies showed that several modern varieties generally considered as representative of citrus basic taxa were introgressed by other species. This was particularly notable for *C*. *maxima* introgressions in mandarins. This observation should explain the low proportion of strong diagnostic SNPs for pummelos and mandarins compared with citrons and *C*. *micrantha*. Therefore, given that our objective was to identify diagnostic SNPs of the basic taxa, it was essential to identify such introgressions in the modern varieties of mandarin, pummelo, citron, and *C*. *micrantha* in order to better estimate the allelic frequencies in the basic taxa. For this purpose, information provided by Factorial Analysis (from dissimilarity values between each pair of accessions) and the estimation of heterozygosity of each genotype was combined for each gene fragment. Genotypes having interspecific phylogeny for the considered fragment were expected to be in intermediate positions between basic taxa clusters and to display much higher heterozygosity than genotypes without interspecific heterozygosity. The C2P27 fragment is provided as an example in Fig 3. For this fragment, four mandarins (‘King’, ‘Dancy’, ‘Ponkan’, and ‘Fuzhu’) displayed clear interspecific heterozygosity. Indeed, while clusters of the basic taxa displayed low heterozygosity (<0.03), these four mandarins had high heterozygosity (average = 0.53), and their intermediate positions between the *C*. *reticulata* and *C*. *maxima* clusters resulted from heterozygosity for SNPs differentially fixed in the *C*. *maxima* and *C*. *reticulata* clusters.

It should be noted that *C*. *micrantha*, which had a relatively central position in the 1/2 plan, had very low heterozygosity and was totally differentiated from the three other basic taxa in the third axis.

Basic taxa allelic frequencies, population diversity parameters (S7 Table) and G_ST_ parameters were then re-estimated, with only gene fragments that did not exhibit interspecific heterozygosity for the considered variety.

**Fig 4:**
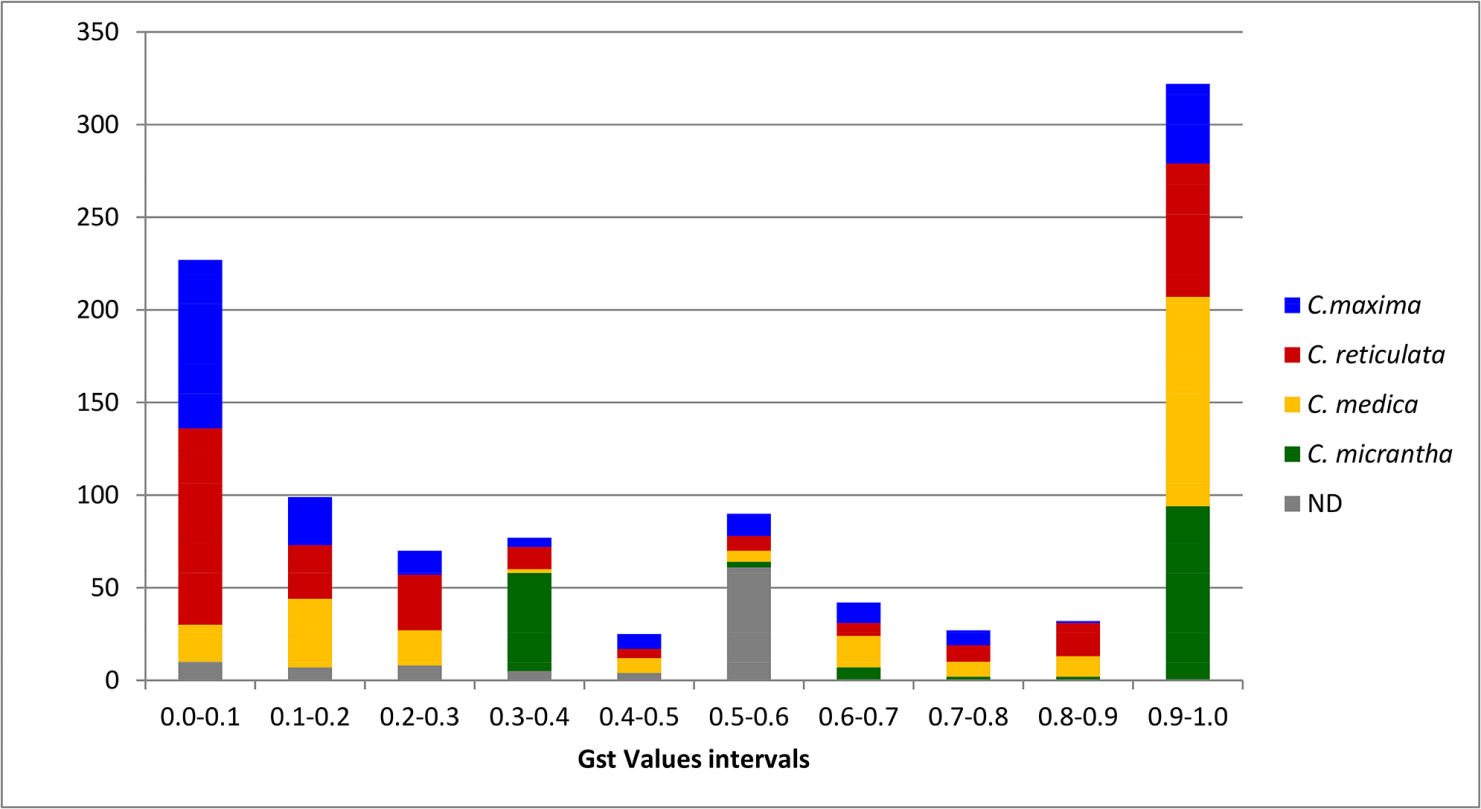
Distribution of SNP G_ST_ values for the four basic taxa from estimated ancestral taxa allelic frequencies after removal of genotypes with interspecific heterozygosity at the gene fragment level.

The distribution of the highest G_ST_ value of each SNP for the four basic taxa (Fig 4) showed that 423 of highest SNP G_ST_ values were >0.6. *C*. *medica* (113 SNPs) and *C*. *micrantha* (92 SNPs) continued to have a larger number of diagnostic SNPs (G_ST_ >0.9) than *C*. *reticulata* or *C*. *maxima*, but the number of strong diagnostic markers for the latter two taxa improved, respectively reaching 72 for *C*. *reticulata* and 43 for *C*. *maxima* (Fig 4). Ninety-five SNPs with a G_ST_ value ≤0.5 did not exhibit any specificity for one of the basic taxa (N.D., Fig 4) and did not meet our diagnostic objectives.

### KASPar marker development

#### SNP marker development

SNPs were selected from those with high differentiation values between basic taxa and those displaying intraspecific variability within each basic taxon (SNPs with fixed allele in three taxa and displaying polymorphism in the fourth taxa). To limit the risk of PCR drift between alleles, SNPs were rejected for KASPar marker development if further SNPs or indels were in close proximity. Eighty-five SNP markers were developed from the 454 SNP mining data. Amplification failed for four of these SNP markers, and eight further SNP regions produced inconsistent results: these twelve markers were thus discarded. The remaining 73 markers were successfully amplified, and according to the 454 genotyping data, 63 of these SNPs were diagnostic for one of the four taxa (12, 9, 20, and 22 markers for *C*. *reticulata*, *C*. *maxima*, *C*. *medica*, and *C*. *micrantha*, respectively). Ten markers characterised intraspecific polymorphisms (two, five, and three markers for *C*. *reticulata*, *C*. *maxima*, and *C*. *medica*, respectively).

#### Conformity between 454 and KASPar genotype calling

Twenty four varieties that had been examined by 454 sequencing were genotyped using the 73 KASPar markers (S4 Table). Of the 73 × 24 genotyping points, 3.6% displayed genotype calling discrepancies between the two methods. The highest discrepancy rate (27%; S2 Fig) was found for the 8p2427684 marker corresponding to the C8P4 gene fragment. However, this region was notable for poor 454 sequencing quality, and data for nine varieties were missing. KASPar data were validated using two technical replicates, and the discrepancies between the two methods were therefore more likely due to 454 genotype calling errors.

#### Transferability of KASPar markers to related genera in the *Aurantioideae* subfamily

Utility of the KASPar markers across the *Aurantioideae* subfamily was assessed using 14 accessions grouped in increasingly distant taxonomic entities according to Swingle and Reece [1]. Missing data and polymorphism information for these different groups are provided in Table 5.

**Table 5:** Screening of SNP markers in different citrus species and subtribes of the *Aurantioideae* subfamily.

	Na	MD	PL	Ho
*Citrus* genus	70	1.0	73	0.05
True citrus *	8	4.3	18	0.02
Near citrus	2	9.6	7	0.05
Primitive citrus	1	12.3	2	0.03
*Triphasilinae*	1	24.7	0	0.00
*Clauseniae*	2	38.4	3	0.03

Na: sample size; MD: missing data (%); PL: number of polymorphic loci; Ho: observed heterozygosity

*True citrus without *Citrus* genus.

The missing data rate was very low in *Citrus* (1.0%) and low in the “true citrus fruit trees” group (4.3%, excluding the *Citrus* genus). The missing data rate increased, respectively, to 9.6% and 12.3% in the “near citrus” and “primitive citrus” groups of the *Citrinae* subtribe. The missing data rate reached a level of 24.7% for the other subtribe of the *Citreae* tribe (*Triphasilinae*) and 38.4% for the two representatives of the *Clauseniae* tribe. These results indicate an increasing loss of transferability with increasing taxonomic distance. As expected due to the discovery panel, the *Citrus* genus was the most polymorphic (73 polymorphic loci), followed by the “true citrus fruit trees” group without the *Citrus* genus (18 polymorphic loci). For some markers (46/73), all accessions except those in the *Citrus* genus displayed the same homozygous genotypes for the non-diagnostic allele. For these 46 markers, the alternative allele fixed in all citrus and relative taxa, with the exception of one of the four basic citrus taxa, was clearly the ancestral allele. The alternative allele therefore resulted from a mutation that occurred during the separation of the differentiated basic taxa.

### Admixture structure of modern citrus varieties

In addition to the 73 new KASPar SNP markers, we selected 32 of our previously developed SNP markers [8,9] to complete the analysis of admixture structure of modern citrus varieties. This selection was made with the same criteria as the new KASPar, based on the available differentiation parameter data between the basic taxa (our unpublished data). Respectively, 30, 23, 30, and 22 of the 105 markers were selected as diagnostic or to represent specific alleles of *C*. *reticulata*, *C*. *maxima*, *C*. *medica*, or *C*. *micrantha*.

**Fig 5:**
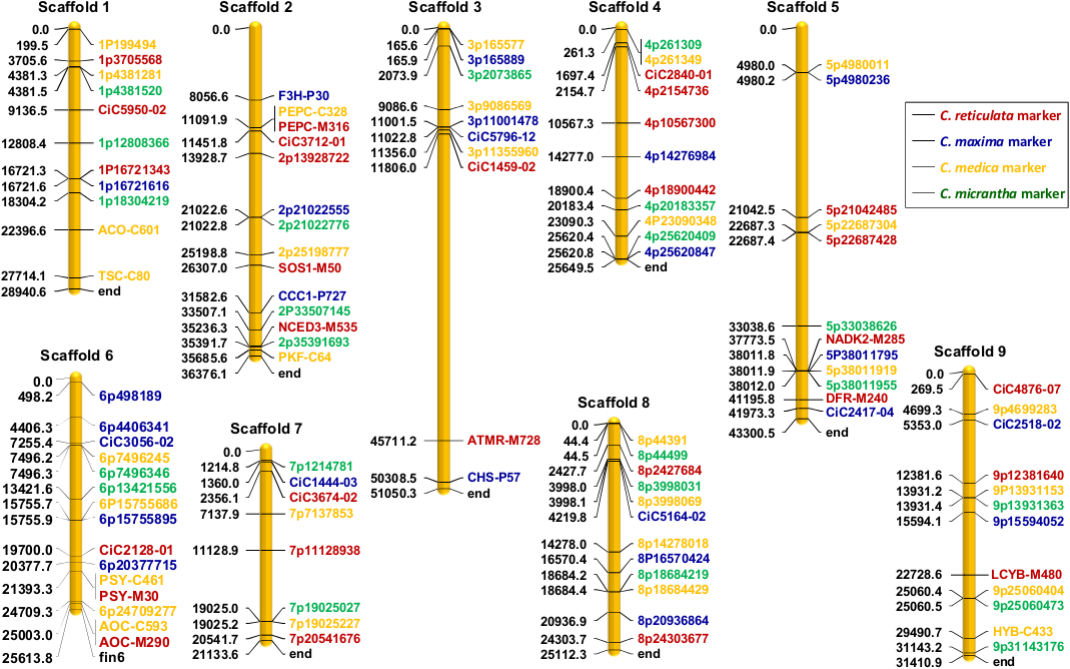
Location of all KASPar SNP markers used. 105 markers; position in kb according to reference sequence of the haploid clementine (Wu *et al*. [20]; Phytozome.net).

The distribution in the clementine reference sequence [20] and allele specificity of the 105 KASPar SNP markers used for Citrus admixture analysis is provided in Fig 5. Specific markers for all basic taxa were present on each chromosome. However, some large lacunas without markers were still present, particularly in chromosomes 3 and 5.

Diversity parameter (Ho and He) and genetic organization parameter (F_w_, F_ST_, and specific G_ST_) values for each marker as well as average values are in S8 Table. With the exception of *C*. *micrantha*, which was totally homozygous and displayed no polymorphisms between the two accessions, the intraspecific parameters Ho and He were not significantly different between the whole set of SNPs identified by 454 and the results of the KASPar analysis.

**Fig 6:**
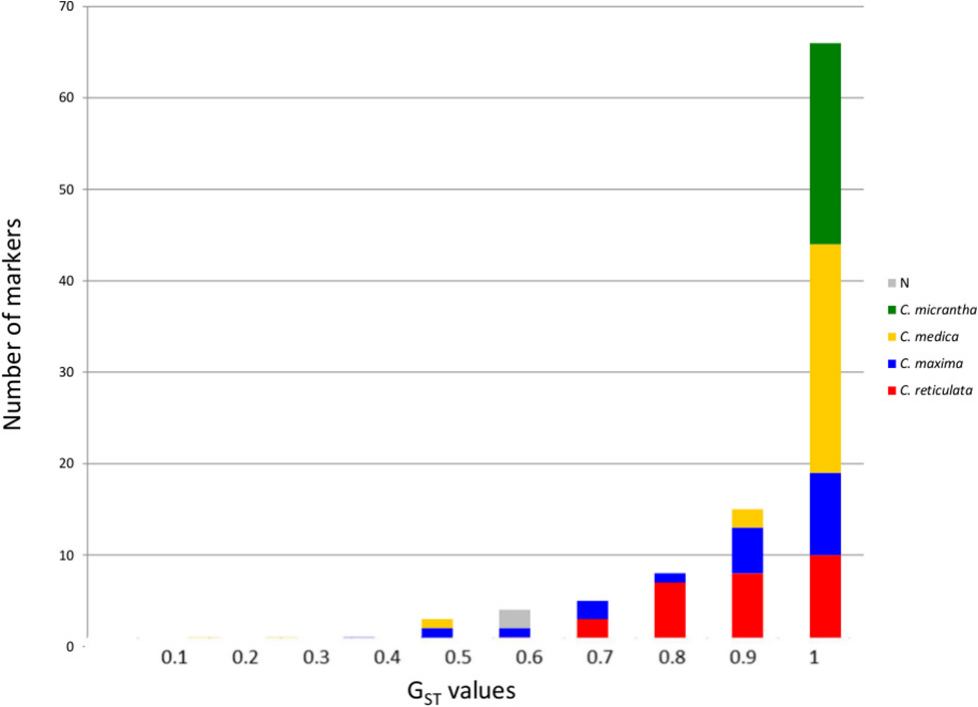
Distribution of individual diagnostic marker G_ST_ values for the four basic taxa through consideration of representatives of the four basic horticultural groups as subpopulations.

However, the structuration of the population was higher with the selected KASPar markers, with average F_w,_ and F_ST_ values of 0.87 ± 0.03, and 0.90 ± 0.03, respectively. The distribution of the G_ST_ parameters (Fig 6) confirmed the efficiency of the applied selection with 18, 14, 27, and 22 markers with a G_ST_ >0.8 for the *C*. *reticulata*, *C*. *maxima*, *C*. *medica*, and *C*. *micrantha* diagnostic markers, respectively. However, two markers (7p11128938 and CiC2518-02) were found to share similar polymorphisms in mandarins and pummelo and were therefore discarded from the specific allele homozygosity/heterozygosity analyses. We therefore concluded that 29, 22, 30, and 22 of the selected markers displayed specific alleles for *C*. *reticulata*, *C*. *maxima*, *C*. *medica*, and *C*. *micrantha*, respectively. Nine of these markers, with lower specific G_ST_ values (<0.6), were fixed for the same allele in three horticultural groups but displayed an intraspecific polymorphism in the fourth group (one, five, and three markers with variant alleles only in mandarin, pummelo, and citron, respectively).

When secondary species accessions were considered (Table 6), no inter-varietal polymorphism was found within *C*. *aurantium* (three accessions), *C*. *sinensis* (three accessions), or *C*. *paradisi*, while four different genotypes were observed in *C*. *aurantifolia* (four accessions) and five in *C*. *limon* (six accessions). All secondary species displayed high heterozygosity values (Ho) compared with the four horticultural groups. Ho ranged from 0.35 in *C*. *paradisi* to 0.48 in *C*. *limon*. The mandarin hybrid, tangor, and tangelo groups displayed increasing average Ho values (0.18, 0.21, and 0.25, respectively). However, substantial heterozygosity variations were observed between varieties within the tangor group (0.13–0.30), and the differences between the groups were thus not statistically significant.

**Table 6:** Diversity of secondary species and modern hybrid varieties assessed using 105 SNP markers.

	N	Ho	NSG
*C*. *aurantium*	3	0.424 ± 0.000	1
*C*. *aurantifolia*	4	0.457 ± 0.020	4
*C*. *limon*	6	0.485 ± 0.045	5
*C*. *paradisi*	3	0.346 ± 0.000	1
*C*. *sinensis*	3	0.371 ± 0.000	1
*C*. *amblycarpa*	1	0.456	1
*C*. *hystrix*	1	0.168	1
Mandarin hybrids	4	0.180 ± 0.021	4
Tangors	8	0.214 ± 0.046	7
Tangelos	3	0.248 ± 0.011	3
Orangelo	1	0.205	1

N: number of accessions by taxa; Ho: observed heterozygosity; NSG: number of single multilocus genotypes.

**Fig 7:**
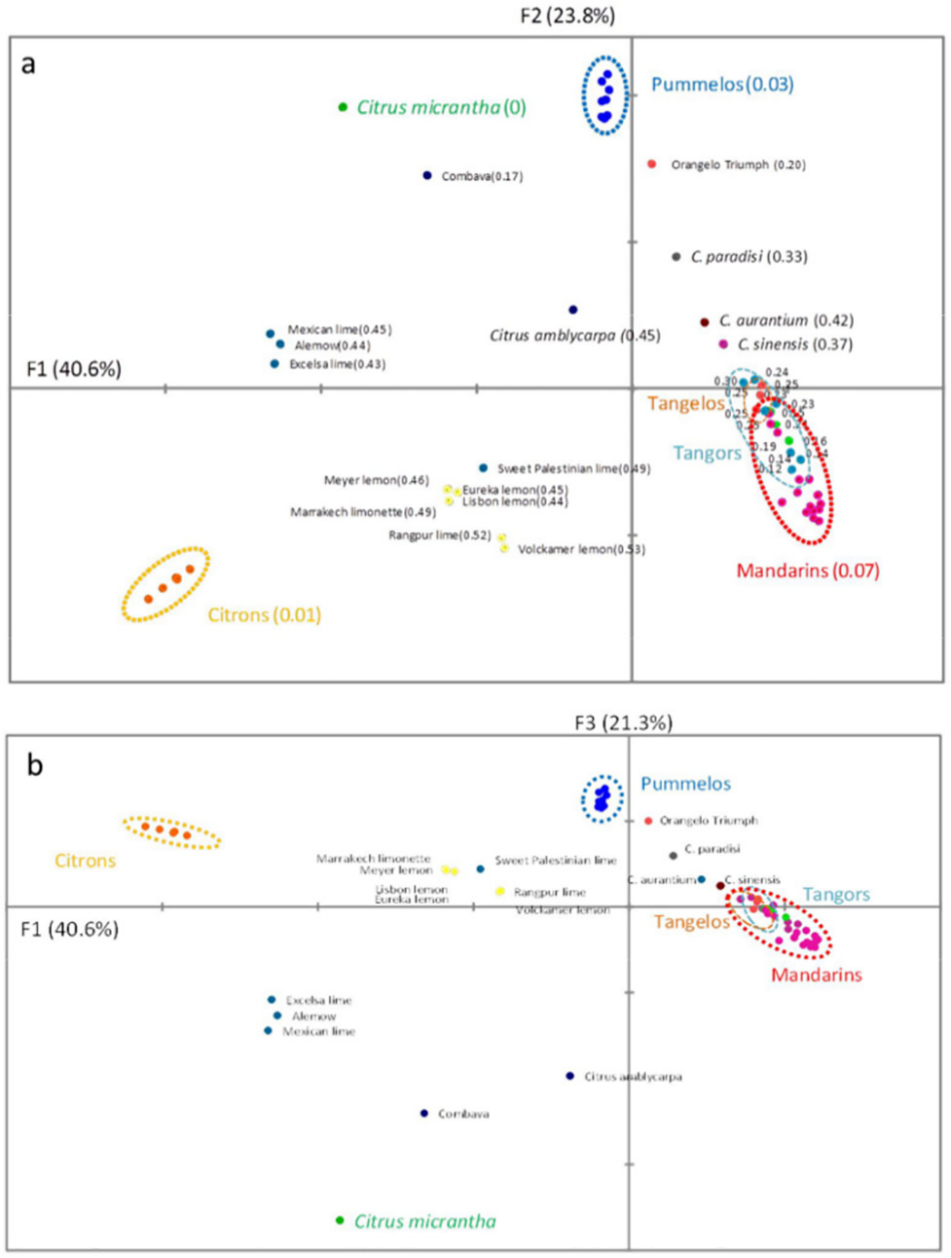
PCA distribution of 70 *Citrus* cultivars from 105 SNP marker genotyping. First three axes. Individual heterozygosity is provided in parentheses after the variety name in the F1/F2 plan. a. F1/F2; b. F1/F3.

PCA was performed with the data from the 70 *Citrus* cultivars genotyped with the 105 KASPar SNPs. The population displayed a very strong structuration, with 85.6% of the total diversity encompassed by the three first axes (40.6%, 23.8%, and 21.3%, respectively). The fourth axis supported only 1.7% of the diversity. The first axis mainly differentiated citrons from other species (Fig 7). The second axis distinguished *C*. *maxima* and *C*. *micrantha* from *C*. *reticulata* and *C*. *medica*, while the third axis separated *C*. *micrantha* for all the other basic taxa. Interestingly, mandarin hybrids, tangors, tangelos, sweet oranges, sour oranges, and grapefruits were distributed along a line between the pummelo and mandarin clusters. Varietal heterozygosity increased from the distal parts of the segment defined by the pummelo cluster (average Ho = 0.03) and the mandarin cluster (average Ho = 0.07) to the central region (Ho = 0.42 for the three accessions of the *C*. *aurantium* sour orange group). ‘Mexican’ lime, ‘Alemow’, and ‘Excelsa’ lime were clustered in intermediary positions between the citron cluster and *C*. *micrantha*, with heterozygosity values of 0.43–0.45. ‘Rangpur’ lime and ‘Volkamer’ lemon also displayed high heterozygosity values (0.52 and 0.53, respectively) and were located between the citron and the mandarin clusters. The ‘Palestinian’ sweet lime, ‘Meyer’ lemon, and ‘Eureka’ lemon were slightly displaced, having higher values for the F2 axis and heterozygosity values of 0.44–0.49. *C*. *amblycarpa* had a similar heterozygosity level but was located between *C*. *micrantha* and the mandarin cluster.

The relative contributions of the ancestral taxa to the modern cultivars suggested by PCA and average heterozygosity levels were confirmed by a deeper analysis of cultivar homozygosity and heterozygosity for the four sets of diagnostic markers (markers with specific alleles for *C*. *reticulata*, *C*. *maxima*, *C*. *medica*, and *C*. *micrantha*).

**Fig 8:**
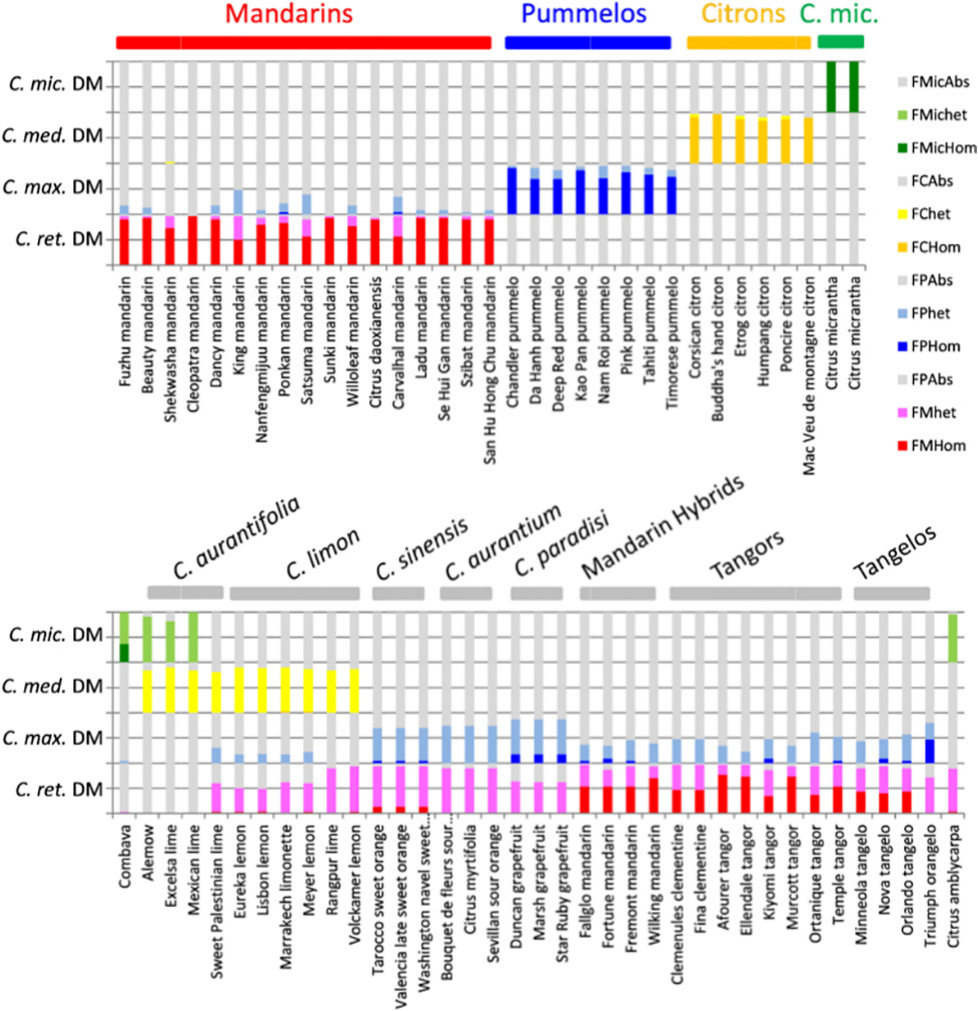
Frequency of specific alleles of the four basic taxa in homozygosity and heterozygosity for 70 *Citrus* accessions analysed with 103 species diagnostic markers. *Taxon* DM: diagnostic markers for the considered taxon; FMHom, FMHet, FMabs, FPHom, FPHet, FPAbs, FCHom, FCHet, FCAbs, FMicHom, FMicHet, and FMicAbs are, respectively, the frequency of homozygous, heterozygous, and absent specific alleles for *C*. *reticulata*, *C*. *maxima*, *C*. *medica*, and *C*. *micrantha*.

Among the four basic horticultural groups, no evidence of interspecific introgression was found for the analysed accessions of pummelos, citrons, and *C*. *micrantha* (Fig 8). Interspecific introgressions of *C*. *maxima* were observed in several mandarins, with higher contributions noted in Satsuma, ‘King’ and ‘Carvalhal’ mandarins. Lower *C*. *maxima* introgressions were found in ‘Beauty’, ‘Nanfengmiju’, ‘Ladu’, ‘Se Hui Gan’, ‘Szibat’ and ‘San Hu Hong Chu’. Diagnostic marker analyses revealed a *C*. *medica* introgression in the ‘Shekwasha’ mandarin. No introgression was found for three mandarins (‘Cleopatra’, ‘Sunki’ and *C*. *daoxianensis*).

Three of the four *C*. *aurantifolia* accessions (‘Alemow’, ‘Excelsa’, and ‘Mexican’ limes) displayed very similar phylogenomic patterns, with close to half contributions from *C*. *medica* and *C*. *micrantha* with most of *C*. *micrantha* and *C*. *medica* diagnostic markers in heterozygosity (Fig 8). The fourth *C*. *aurantifolia* accession (‘Palestinian’ sweet lime) had a distinct pattern and appeared to have a similar phylogenetic structure to four of the six accessions of *C*. *limon* (‘Meyer’, ‘Eureka’, and ‘Lisbon’ lemons, and ‘Marrakech’ *limonette*). These contained close to 50% *C*. *medica* (most *C*. *medica* diagnostic markers in heterozygosity) and displayed a three ancestral taxa admixture pattern (*C*. *medica*, *C*. *reticulata*, and *C*. *maxima*) with a greater contribution from *C*. *reticulata* than *C*. *maxima*. Diagnostic alleles of *C*. *medica*, *C*. *maxima*, and *C*. *reticulata* were found systematically in heterozygosity in these five accessions. The two last *C*. *limon* accessions (‘Rangpur’ lime and ‘Volkamer’ lemon) had a very similar phylogenetic structure with a complete heterozygosity for their *C*. *reticulata* and *C*. *medica* diagnostic alleles and no diagnostic alleles for the other taxa (Fig 8).

All accessions of *C*. *sinensis*, *C*. *aurantium*, *C*. *paradisi*, mandarin hybrids, tangors, tangelos, and orangelo had a two ancestral taxa admixture structure (*C*. *reticulata* and *C*. *maxima*) with variable contributions. The three *C*. *aurantium* accessions displayed around half contribution of each taxon and appeared totally heterozygous for their *C*. *reticulata* and *C*. *maxima* diagnostic alleles. *C*. *sinensis* had a more complex genomic structure and part of the diagnostic alleles for *C*. *reticulata* and for *C*. *maxima*, were in homozygosity. *C*. *paradisi* accessions had higher contributions of *C*. *maxima* than *C*. *reticulata* with homozygous diagnostic alleles of *C*. *maxima* in addition to heterozygous alleles from *C*. *maxima* and *C*. *reticulata*. All mandarin hybrids, tangors, and tangelos had a greater contribution from *C*. *reticulata* than *C*. *maxima*. All displayed homozygous diagnostic alleles of mandarin in relatively high proportion (34% in ‘Kiyomi’ to 77% in ‘‘Nadorcott’) as well as heterozygous diagnostic alleles of *C*. *reticulata* and *C*. *maxima*. In addition, seven accessions presented one or two *C*. *maxima* diagnostic alleles in homozygosity. The ‘Triumph’ orangelo displayed a similar pattern to *C*. *paradisi* but with a higher proportion of homozygosity for *C*. *maxima* diagnostic alleles (29% of the total number of *C*. *maxima* diagnostic alleles).

*C*. *amblycarpa* displayed around 50% contributions from *C*. *reticulata* and *C*. *micrantha* and appeared totally heterozygous for its *C*. *reticulata* and *C*. *micrantha* diagnostic alleles.

The direct analysis of ‘Combava’ (*C*. *hystrix*) with basic diagnostic SNPs only testified to *C*. *micrantha* specific alleles in homozygosity and heterozygosity without any specific alleles for the three other basic taxa.

Probably some of the strong hypotheses of the Bayesian estimation performed by the Structure software [46] such as Hardy-Weinberg genotypic proportions and no linkage disequilibrium should not be encountered in the considered taxa pertaining to their reproductive mode (contribution of asexual reproduction), however we performed a Structure analysis (S1 File) to check if the estimated contributions based on the species diagnostic marker set should fit with the direct estimation of homozygosity/héterozygosity and PCA analysis. Very low variability in estimated contributions was observed between the ten runs for K = 4 (best model) and Structure identified the four basic taxa as the four parental groups. The average contribution values of the ten runs were globally in agreement with the two previous analysis.

## Discussion

### Amplicon 454 sequencing was an efficient approach for species-diagnostic SNP mining in *Citrus* and for competitive allele-specific PCR marker development

SNPs have become the most abundant and powerful polymorphic codominant markers that can be identified and characterised across whole genomes [52]. SNPs allow the development of very dense genetic linkage maps in animals and plants [53–55]. Moreover, SNPs are generally considered to have a high identity by descent rate and thus are useful for phylogenetic and genetic association studies [56,57]. The primary limitation of SNP markers for gene-pool diversity analysis is that the revealed genetic organisation of the genotyped germplasm is strongly dependent on the discovery panel and the selection strategy used to develop a set of markers from all identified SNP positions [58–63]. This ascertainment bias is particularly notable when SNPs are selected from only one sequenced heterozygous genotype. Examples include SNP characterisation in *Vitis vinifera* L., which used the whole genome sequence of the cultivar ‘Pinot Noir’ [64], and in *Citrus*, which used BAC-end sequencing data from the ‘Clemenules’ clementine [8]. Moreover, unexpected alleles may exist at the target genomic regions. These unknown or ‘null’ alleles can interfere with accurate genotyping of the expected alleles and potentially impact genetic studies in a negative manner [65]. The frequencies of these null alleles are likely to be higher when genotyping samples have wider genetic distances with the discovery panel. Indeed, additional polymorphisms in the genome area targeted by the PCR primers should results in PCR failure.

Next-generation technologies such as 454 amplicon sequencing present affordable opportunities to reduce genome complexity to well-dispersed gene fragments and provide information in approximately 500 pb read sequences. This allows extending greatly the discovery panel compared with a WGS approach. With respect to our objective of developing species-diagnostic PCR markers, the relatively long length of reads presented two advantageous features. First, multilocus sequences were produced, which were more powerful for inference of phylogenetic origins than single-locus sequences. Thus, in this study, combining the comparative average heterozygosity of the amplicons between the different accessions with factorial analysis allowed the identification of mandarin varieties with interspecific heterozygosity for the considered fragment. Therefore the identification of diagnostic alleles for these taxa was improved. Second, a decisive advantage is that it allows selecting diagnostic SNP position without close additional polymorphism that should affect the competitive allele-specific PCR of the developed KASPar marker.

The *Citrus* genus had favourable genetic organization for the identification of diagnostic markers from 500 pb amplicon sequences. The global SNP average rate was 36.7 SNP/kb (1,053 SNPs identified from 28,507 kb), and the average number of SNPs between varieties of two horticultural groups was 10.0–13.19 SNP/kb. As might be expected, this global SNP rate was lower than the 52.9 SNP/kb, reported by Garcia-Lor *et al*. [10], achieved by Sanger sequencing of 27 gene fragments in four related genera (*Poncirus*, *Fortunella*, *Microcitrus*, *and Eremocitrus*) and *Citrus*. However, the authors noted values similar to those reported here for the average number of SNPs between varieties of pummelos, citrons, and mandarins. At the intraspecific level, the higher diversity in *C*. *reticulata* and *C*. *maxima* than in *C*. *medica* is in agreement with previous studies [7,8,10]. Moreover, the high structuration of the diversity among *C*. *maxima*, *C*. *medica*, *C*. *reticulata*, and *C*. *micrantha* agreed with previous molecular studies [4–6,10,66] and numerical taxonomies based on phenotypic traits [13] that recognised these taxa as the ancestors of the cultivated *Citrus* species. As a consequence of the high genetic differentiation resulting from the allopatric evolution of the four basic taxa, 271 of the 1,053 SNPs were found to be highly diagnostic for one of the four basic taxa (specific G_ST_ >0.9).

### The SNP marker set revealed the phylogenetic origins and admixture genomic structures of modern citrus cultivars and rootstocks

#### Raw quantifications of the contributions of four ancestral taxa to modern varieties were coherent with previously estimated values from WGS data in seven genotypes

Recently, Wu *et al*. [20] analysed the phylogenomic structure of several citrus varieties from WGS data. This analysis revealed *C*. *maxima* introgression in two mandarin varieties (‘Ponkan’ and ‘Willowleaf’) that were generally considered as true representatives of *C*. *reticulata*. In addition, the proportion of the *C*. *maxima* genome was quantified in these two cultivars, one mandarin hybrid (‘W. Murcott’ = ‘Nadorcott’), clementine, sweet orange, and sour orange. Interestingly, the values found in our study from Structure analysis based on a limited set of markers (103) were well correlated with the previous observations from WGS data [20]. Indeed, Wu *et al*. [20] found decreasing proportions of *C*. *maxima* from sour orange to ‘Willowleaf’ as follows: sour orange (0.49), sweet orange (0.44), clementine (0.21), ‘W. Murcott’ (0.15), ‘Ponkan’ (0.077), and ‘Willowleaf’ (0.045). Our structure analysis inferred values of 0.509, 0.391, 0.236, 0.125, 0.035, and 0.040, respectively, for the same varieties. For the seven cultivars the correlation coefficients for estimations of *C*. *maxima* and *C*. *reticulata* contributions in the two studies were very high (0.993). Moreover, the analysis of the proportions of the diagnostic alleles of *C*. *reticulata* and *C*. *maxima* in heterozygosity and homozygosity agreed with the Wu *et al*. [20] analysis, with only heterozygous diagnostic alleles for sour orange, homozygous and heterozygous alleles of both taxa for sweet orange, and only specific *C*. *maxima* alleles in heterozygosity in ‘Willowleaf’, ‘Nadorcott’, and clementine. Therefore, we consider that the estimations of interspecific genomic structure provided by the set of species-diagnostic markers constitute a first approximation of the true phylogenomic structures of the analysed genotypes. As a result of their selection process, these markers minimised intraspecific variability. The SNP markers should therefore be combined with a set of SSR markers to retain intraspecific variability data when analysing germplasm diversity. Indeed, in citrus, comparative studies of SSRs, indels, and SNP markers [7,29] showed that SSRs were the more powerful tool for analysis of intraspecific variability.

#### Numerous old and modern mandarin, tangor, tangelo and orangelo varieties were introgressed by *C*. *maxima*

The representative genotypes of the pummelo and citron horticultural groups appeared, respectively, as pure *C*. *maxima* and *C*. *medica* without identified interspecific introgressions. Similarly, no evidence of introgression was found in the only two *C*. *micrantha* observed. As mentioned above, based on WGS analysis, Wu *et al*. [20] evidenced introgressions of *C*. *maxima* in two mandarin varieties considered as true representatives of *C*. *reticulata*. A 454 amplicon haplotype study for chromosome 2 [51] also revealed introgression by *C*. *maxima* in nine of the thirteen mandarins studied. In the present work, species-diagnostic marker analysis revealed such *C*. *maxima* introgression in 13 of the 17 mandarin analysed. Only three mandarins were found without any indication of interspecifi*c* introgression (‘Cleopatra’, ‘Sunki’, and *C*. *daoxianensis*). *C*. *daoxianensis* is considered as an ancestral mandarin [67], and the two others are mostly used as rootstock. From cytoplasmic analysis with mitochondrial indels (our unpublished data), it appears that these three mandarins share the acidic mandarin mitotype defined previously [68], while all mandarins found introgressed by *C*. *maxima* share the mitotype of edible mandarins [68] and are cultivated for fruit consumption. Therefore, the reticulation(s) event(s) between *C*. *reticulata* and *C*. *maxima* and further introgression processes appear to be important components of the mandarin domestication. Only deep genomic analysis will determine whether all edible mandarins result from one or several reticulation events and how introgression produced the modern mandarin displaying only a limited part of the *C*. *maxima* genome. During the 20^th^ century, mandarin breeding was based on sexual hybridisations between mandarins but also between mandarins and sweet oranges (tangors), and mandarins and grapefruits (tangelos). All these recent hybrids, as well as supposed natural tangors such as ‘Ortanique’, ‘Murcott’, ‘Temple’, ‘Nadorcott’, and clementine, displayed admixture structure genomes between *C*. *reticulata* and *C*. *maxima* with predominant contribution of *C*. *reticulata*. It appears that mandarins, tangelos, tangors, sweet oranges, sour oranges, grapefruits, and the orangelo (grapefruit × sweet orange hybrid) provide a large range of phylogenomic constitutions between the *C*. *reticulata* and *C*. *maxima* clusters. This is favourable for genetic association studies based on phylogenomic structures of the germplasm.

#### The phylogenetic origin of secondary species is confirmed or revealed

The partial apomixis of most of the secondary species explained that, in agreement with previous molecular studies [6–8,22], no polymorphisms were found between the analysed accessions within *C*. *sinensis*, *C*. *aurantium*, and *C*. *paradisi*, although they were highly heterozygous (Ho of 0.371, 0.424, and 0.346, respectively, for the 105 KASPar SNP markers). This confirms that the intraspecific phenotypic polymorphisms in these secondary species arose from punctual mutation, transposable element movement [69], or epigenetic variation rather than sexual recombination. Conversely, intervarietal variability was found for most of the analysed *C*. *limon* and *C*. *aurantifolia* accessions with the exception of ‘Eureka’ and ‘Lisbon’ lemons.

*C*. *sinensis and C*. *aurantium* are believed to derive from hybridisations between the *C*. *maxima* and *C*. *reticulata* gene pools [5,6,8,10,20,70]. Previous SSR marker studies [7,71] and a SNP study using a narrow discovery panel [8] suggest that predominant portions of their genomes arose from the *C*. *reticulata* gene pool. The present study concurred with the conclusions obtained from WGS data [20]. In PCA, sour orange displayed an intermediary position between the pummelos and the mandarin group. It was highly heterozygous for *C*. *maxima* and *C*. *reticulata* specific alleles and structure analysis inferred close to 50% contribution for each of the two species. This is in agreement with a direct hybridisation between *C*. *maxima* and *C*. *reticulata* as proposed by Garcia-Lor *et al*. [10] and Wu *et al*. [20]. Sweet orange appeared to have developed from a more complex combination between two parents already displaying admixture structure between *C*. *reticulata* and *C*. *maxim*a, as testified by the presence of specific *C*. *maxima* and *C*. *reticulata* alleles in homozygosity.

*C*. *paradisi*: the origin of grapefruit is attributed to a natural hybridisation between pummelo (*C*. *maxima*) and sweet orange (*C*. *sinensis*). This hybridisation may have occurred in the Caribbean more than 200 years ago [13,72,73]. In this study, grapefruit had an intermediary position between the sweet orange and pummelo gene pools in the PCA representation. *C*. *reticulata* specific alleles were displayed in heterozygosity and *C*. *maxima* allele were displayed in heterozygosity or in homozygosity. Our results are therefore consistent with the pummelo (*C*. *maxima*) x sweet orange (derived from hybridisations between the *C*. *maxima* and *C*. *reticulata* gene pools) hybridisation hypothesis.

*C*. *aurantifolia*: Tanaka [2] considered ‘Mexican’ lime, ‘Excelsa’ lime, ‘Alemow’, and ‘Palestinian’ sweet lime as four distinct species, namely, *C*. *aurantifolia*, *C*. *excelsa*, *C*. *macrophylla*, and *C*. *limettioïdes*, respectively. However, Swingle and Reece [1] combined these within *C*. *aurantifolia*. In all of the analyses reported here, the three first genotypes displayed limited differences, whereas ‘Palestinian’ sweet lime appeared much more related to several *C*. *limon* cultivars. In PCA, the ‘Mexican’ lime, ‘Excelsa’ lime, and ‘Alemow’ displayed an intermediary position between the citron cluster and *C*. *micrantha*. These were highly heterozygous for *C*. *micrantha* and *C*. *medica* specific alleles, and structure analysis inferred close to 50% contribution for each of the two species. Therefore, our results suggest a similar origin by direct hybridisation between *C*. *micrantha* and *C*. *medica* for these three varieties. For ‘Mexican’ lime, this agrees with the hypothesis proposed by Nicolosi *et al*. [5]. Froelicher *et al*. [68] showed that ‘Mexican’ lime and ‘Alemow’ share the *C*. *micrantha* mitotype. Recent analysis with mitochondrial indels and chloroplatic SSRs [74] leads to the same conclusion for ‘Excelsa’ lime. Therefore, ‘Mexican’ lime, ‘Excelsa’ lime, and ‘Alemow’ clearly have similar papeda × *C*. *medica* origins. An enhanced study of papeda germplasm will be necessary to definitively conclude *C*. *micrantha* or another papeda as the female parent of these three varieties. ‘Palestinian’ sweet lime structure will be discussed with *C*. *limon*.

*C*. *limon*: ‘Eureka’/’Lisbon’ lemon, ‘Marrakech’ limonette, ‘Meyer’ lemon, ‘Rangpur’ lime, ‘Volkamer’ lemon were considered by Tanaka [2] as four species, respectively, *C*. *limon*, *C*. *limetta*, *C*. *meyeri*, and *C*. *limonia*. These four species were joined in *C*. *limon* by Swingle and Reece [1]. Our analysis clearly distinguished two main groups of admixture structure. The first was comprised of the *C*. *limon*, *C*. *limetta*, and *C*. *meyeri* species as defined by Tanaka. All displayed a three species admixture structure (*C*. *medica*, *C*. *reticulata*, and *C*. *maxima*) with specific alleles of these three taxa in heterozygosity. The ‘Palestinian’ sweet lime (*C*. *limettioïdes*) displayed a very similar pattern. The contribution of *C*. *medica*, as revealed by structure analysis, was close to 50% for all these varieties. Therefore, they are probably direct hybrids between *C*. *medica* and varieties with admixture structure between *C*. *maxima* and *C*. *reticulata*. Based on RFLP, RAPD, and CAPS data, Nicolosi *et al*. [5] were the first to propose that “yellow lemons” arose from a hybridisation between *C*. *aurantium* and *C*. *medica*. This hypothesis was supported by nuclear SSR [7,74] and SNP [8] analyses as well as mitochondrial research [68], and is coherent with the present results for ‘Eureka’ and ‘Lisbon’ lemon. The two *C*. *limonia* accessions (‘Volkamer’ lemon and ‘Rangpur’ lime) shared similar characteristics that differed from the previous lemon and lime patterns. In PCA, the *C*. *limonia* accessions displayed an intermediary position between *C*. *medica* and the mandarin group. The accessions were heterozygous for most *C*. *medica* and *C*. *reticulata* specific alleles, and structure analysis inferred close to 50% contributions from each of the two species. Therefore, *C*. *limonia* accessions results probably from direct hybridizations between *C*. *reticulata* and *C*. *medica*. Previous mitochondrial marker analyses [68] showed that ‘Volkamer’ lemon and ‘Rangpur’ lime shared the cytoplasm of acid mandarins that would be expected as the maternal parents of the two *C*. *limonia* accessions.

*C*. *amblycarpa* is native to Indonesia, where it is called Djerook leemo [75]. It is generally considered to be a mandarin hybrid, and its common English name is ‘Nasnaran’ mandarin. However, Froelicher *et al*. [68] showed that it has a papeda mitotype, identical to *C*. *micrantha* and *C*. *hystrix*. In PCA, *C*. *amblycarpa* displayed an intermediary position between *C*. *micrantha* and the mandarin group. It was highly heterozygous for *C*. *micrantha* and *C*. *reticulata* specific alleles, and structure analysis inferred close to 50% contribution from each of the two species. Therefore, the hypothesis of papeda × acidic mandarin proposed for *C*. *amblycarpa* by Ollitrault *et al*. [8] was confirmed.

## Conclusions

The 454 sequencing of 57 gene fragments covering the nine chromosomes of the haploid citrus set for 26 genotypes revealed that the length of 454 reads and the level of differentiation between the ancestral taxa of modern citrus allowed efficient selection of ancestral species-diagnostic markers. A large number (271) of the 1,053 SNPs mined from the 28,507 kb of amplicon sequence displayed specific G_ST_ values >0.9 for one of the basic taxa. Seventy-three KASPar markers were successfully developed and used with 32 previously developed SNP markers for analysis of the admixture structure of actual varieties and rootstock. Good correlations were observed between the contribution of the four basic taxa inferred with the set of species-diagnostic markers and recent published data from WGS of seven citrus varieties. The analysis of admixture genomic structures of cultivated citrus species and cultivars with 105 species-SNP diagnostic markers revealed *C*. *maxima* introgressions in most modern mandarin cultivars and in all recent selections of small citrus issued from 20^th^ century breeding programs. This suggests that *C*. *reticulata* × *C*. *maxima* reticulation events and introgression processes were important elements of sweet mandarin domestication. The large range of phylogenomic constitutions between *C*. *reticulata* and *C*. *maxima*, revealed in modern mandarin, tangelo, tangor, sweet orange, sour orange, grapefruit, and orangelo germplasm, appears to be favourable for genetic association studies based on phylogenomic structures of the germplasm. Inferred admixture structures of several secondary citrus species were in agreement with previous hypotheses regarding their origin. Admixture structures also revealed the genomic structure and probable origin of several acid citrus varieties (‘Excelsa’ lime, ‘Rangpur’ lime, ‘Alemow’, ‘Marrakech’ Limonette, ‘Palestinian’ sweet lime, and ‘Volkamer’ lemon) and the incorrect assignation of ‘Palestinian’ sweet lime to *C*. *aurantifolia* by Swingle and Reece [1]. The developed species-diagnostic SNP marker set will be very useful for systematic estimation of admixture structure of the citrus germplasm. In addition, the marker set will find many applications in citrus genetics for genetic mapping of secondary species, analysis of meiotic mechanisms (disomic/tetrasomic inheritance) in double-diploid secondary species, and study of the origin of 2n gametes in interspecific admixture genotypes, as well as for more routine activities such as nucellar/zygotic discrimination.

## Supporting Information

S1 Fig Distribution of the highest SNP G_ST_ values for the four basic horticultural groups (454 data from 26 cultivars). N.D.: non-diagnostic.
(TIF)

S2 Fig Distribution of the markers displaying discrepancies between 454 and KASPar genotype calling. x axis: discrepancy rate; y axis: number of markers.
(TIF)

S1 File Structure analysis from 103 species diagnostic SNP markers. Table A, Estimation of the best number of subpopulation in Structure software analysis based on 103 SNP markers. Table B, Means and confidence interval of the contribution of the four basic taxa estimated from 103 SNP markers (from 10 permuted and aligned independent Structure software run cluster outputs). Figure A, Structure analysis of 70 *Citrus* cultivars from genotyping data with 103 SNP markers. Red, blue, yellow, and green correspond to the inferred contributions of *C*. *reticulata*, *C*. *maxima*, *C*. *medica*, and *C*. *micrantha*, respectively.
(PDF)

S1 Table Accession list. (XLSX)

S2 Table Multiplex genotype Identifiers (MID) and related genotypes. (XLSX)

S3 Table Selected gene fragments and associated primers. (XLSX)

S4 Table SNP marker information. (XLSX)

S5 Table Diversity and structuration parameter values for 1053 SNPs identified in 26 cultivars representative of the four basic horticultural groups. (XLSX)

S6 Table SNP/kb for each gene fragment. (XLSX)

S7 Table Diversity of citrus basic taxa after removal of genotypes with interspecific heterozygosity at the gene fragment level. (XLSX)

S8 Table Diversity parameters of the 105 KASPar SNP markers within and between the four basic horticultural groups. (XLSX)
