# Supplementary figures and images for: Nuclear Species-Diagnostic SNP Markers Mined from 454 Amplicon Sequencing Reveal Admixture Genomic Structure of Modern Citrus Varieties
Source: PLoS One. 2015 May 14;10(5):e0125628. doi: 10.1371/journal.pone.0125628 (PMC4431842; doi:10.1371/journal.pone.0125628)

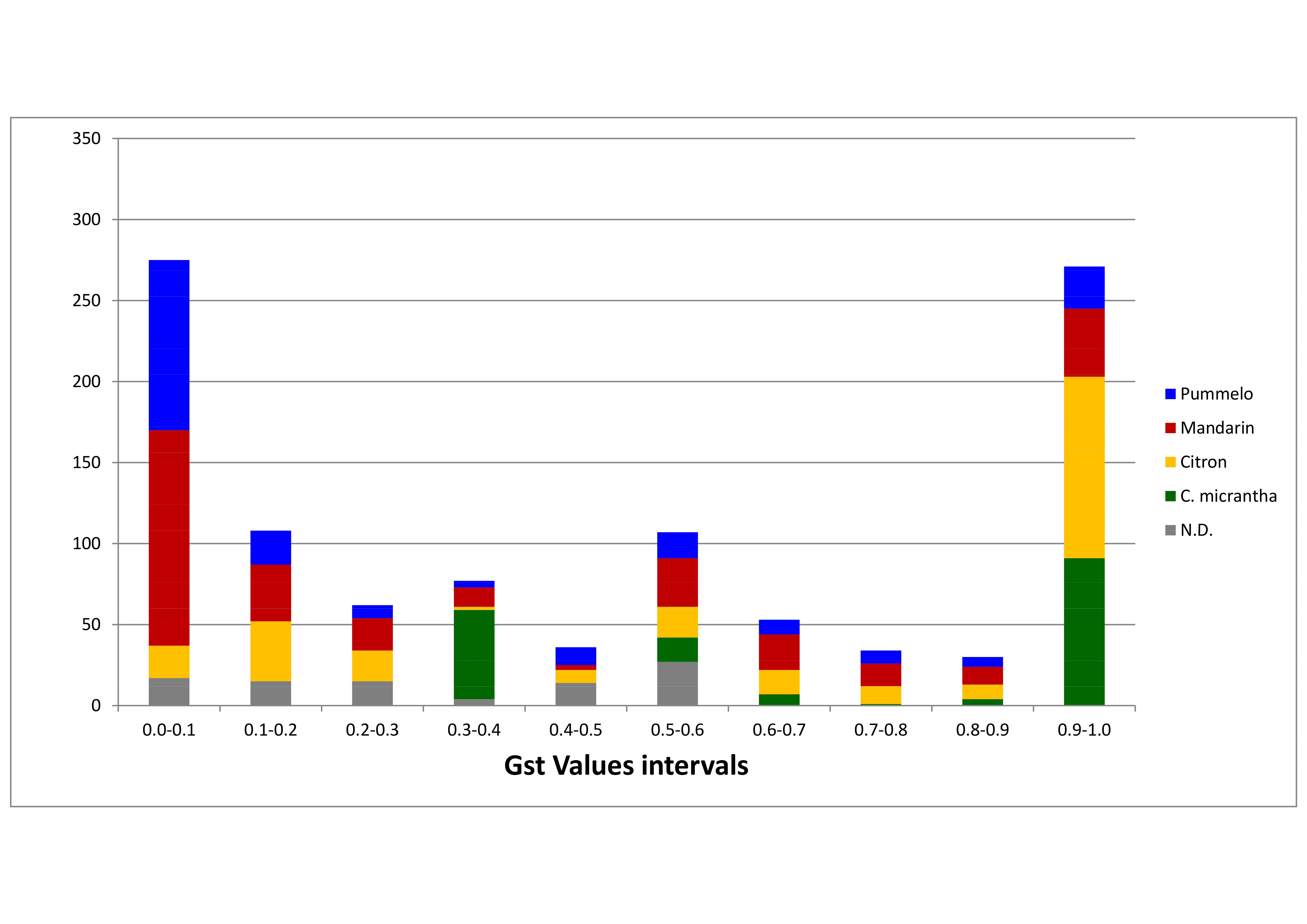

Supplement: S1 Fig — N.D.: non-diagnostic. (TIF) [file pone.0125628.s001.tif]

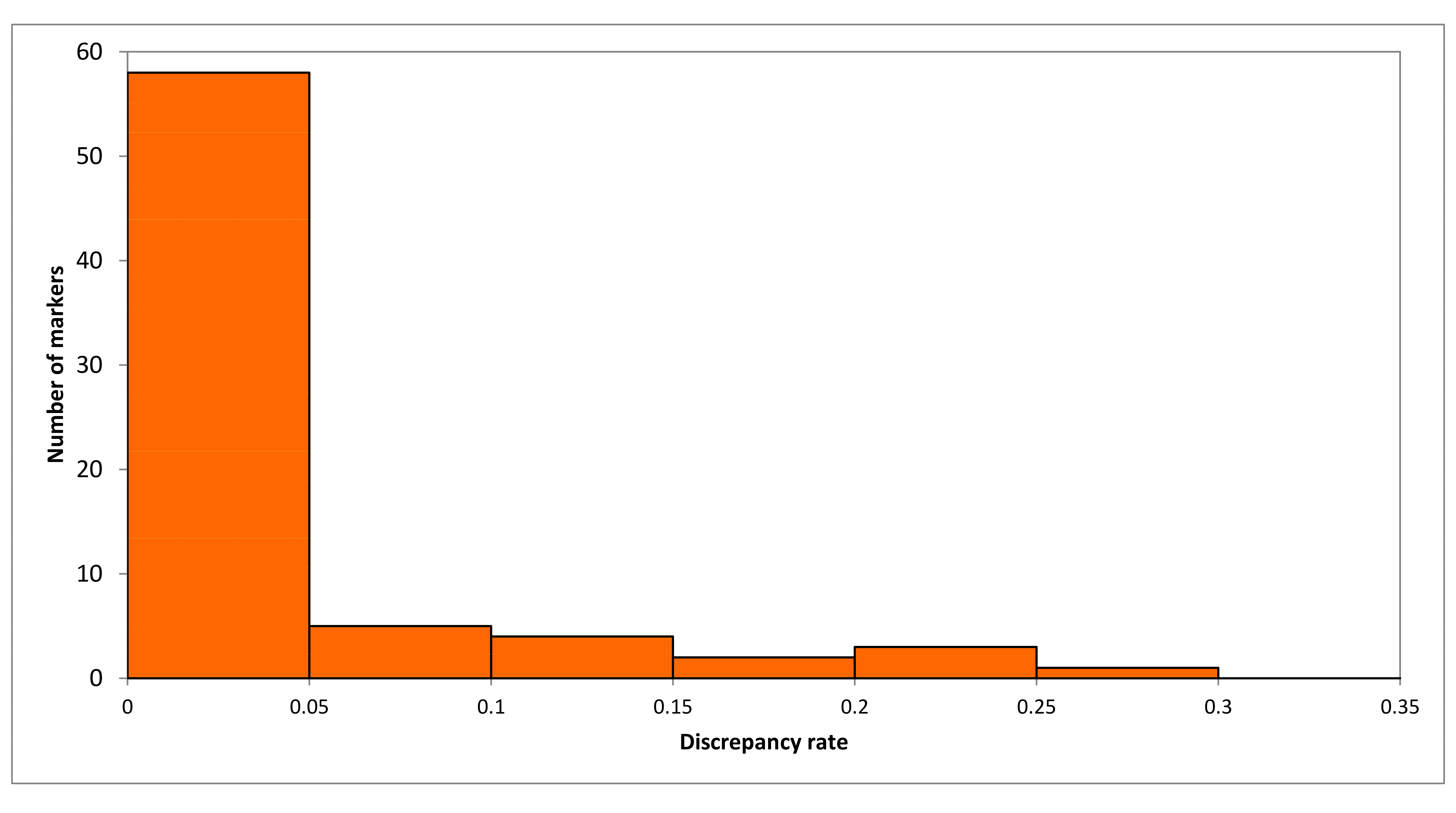

Supplement: S2 Fig — x axis: discrepancy rate; y axis: number of markers. (TIF) [file pone.0125628.s002.tif]
