# Supplementary material for: Nuclear Species-Diagnostic SNP Markers Mined from 454 Amplicon Sequencing Reveal Admixture Genomic Structure of Modern Citrus Varieties
Source: PLoS One. 2015 May 14;10(5):e0125628. doi: 10.1371/journal.pone.0125628 (PMC4431842; doi:10.1371/journal.pone.0125628)
Supplement: S1 File — Table A, Estimation of the best number of subpopulation in Structure software analysis based on 103 SNP markers. Table B, Means and confidence interval of the contribution of the four basic taxa estimated from 103 SNP markers (from 10 permuted and aligned independent Structure software run cluster outputs). Figure A, Structure analysis of 70 Citrus cultivars from genotyping data with 103 SNP markers. Red, blue, yellow, and green correspond to the inferred contributions of C. reticulata, C. maxima, C. medica, and C. micrantha, respectively. (PDF) [file pone.0125628.s003.pdf]

Supplementary Information S1 Pdf File: Structure analysis from 103 species diagnostic SNP markers

Table A: Estimation of the best number of subpopulation in structure analysis based on 103 SNP markers.

Population structure was inferred using the Structure program version 2.3.4 (Pritchard Lab, 2014). No a priori population structure was defined. The linkage model option was used, with allele frequencies correlated and computed probability of the data for K estimating. Analyses were made with K value (number of subpopulations) varying from 1–10. The statistics used to select the correct K value were as in Evanno *et al.* (2005): the mean likelihood, L(K); the mean difference between successive likelihood values of K, L'(K); the absolute value of this difference, °L''(K)°; and ΔK, which is the mean of the absolute values of L''(K) divided by the standard deviation of L(K). The likelihood distribution L(K) and ΔK were the main values used to choose the optimal K value of the population. Analysis of delta K indicated that optimal results were obtained with K = 4.

	MEDIA	DESVEST	L'(K)	IL''(K)I	ΔK
K=1	-6889.08	0.14757			
K=2	-4895.27	6.56236	1993.81	819.56	124.888017
K=3	-3721.02	38.9405	1174.25	509.27	13.0781418
K=4	-3056.04	1.19555	664.98	626.73	524.220165
K=5	-3017.79	2.26296	38.25	10.42	4.60458327
K=6	-2989.96	18.6591	27.83	14.29	0.76584788
K=7	-2976.42	17.5488	13.54	14.03	0.79948594
K=8	-2948.85	18.2035	27.57	13.84	0.76029357
K=9	-2935.12	20.7344	13.73	2.25	0.10851511
K=10	-2923.64	13.4779	11.48	11.48	0.85176659

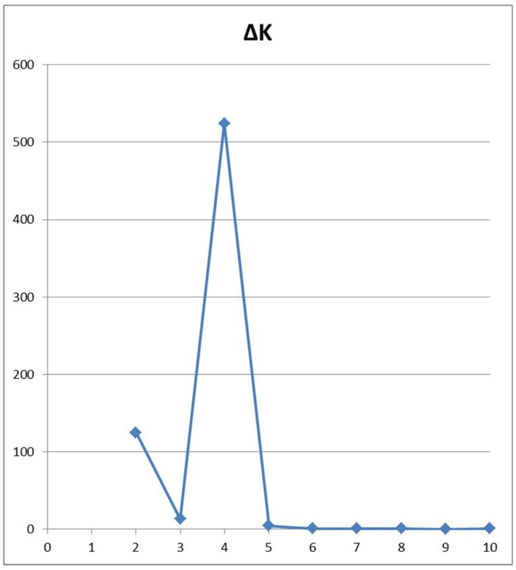

Table B: Means and confidence interval of the contribution of the four basic taxa estimated from 103 SNP markers (from 10 permuted and aligned independent Structure software run cluster outputs)

Genotype	Missing Data (%)	*C. micrantha*	Citron	Mandarin	Pummelo
Fuzhu mandarin	0	0.003 ± 0.000	0.002 ± 0.000	0.963 ± 0.000	0.030 ± 0.000
Beauty mandarin	0	0.002 ± 0.000	0.003 ± 0.000	0.991 ± 0.000	0.003 ± 0.000
Shekwasha mandarin	0	0.006 ± 0.000	0.051 ± 0.000	0.931 ± 0.000	0.010 ± 0.000
Cleopatra mandarin	0	0.003 ± 0.000	0.003 ± 0.000	0.992 ± 0.000	0.002 ± 0.000
Dancy mandarin	0	0.003 ± 0.000	0.002 ± 0.000	0.963 ± 0.000	0.030 ± 0.000
King mandarin	0	0.012 ± 0.001	0.004 ± 0.000	0.750 ± 0.002	0.234 ± 0.002
Nanfengmijuu mandarin	0	0.004 ± 0.000	0.004 ± 0.000	0.984 ± 0.000	0.006 ± 5.666
Ponkan mandarin	0	0.003 ± 0.000	0.002 ± 0.000	0.958 ± 0.000	0.035 ± 0.000
Satsuma mandarin	0	0.003 ± 0.000	0.003 ± 0.000	0.722 ± 0.002	0.269 ± 0.002
Sunki mandarin	0	0.003 ± 0.000	0.002 ± 0.000	0.991 ± 0.000	0.003 ± 0.000
Willoleaf mandarin	1	0.003 ± 0.000	0.002 ± 0.000	0.953 ± 0.000	0.040 ± 0.000
Citrus daoxianensis	3	0.002 ± 0.000	0.002 ± 0.000	0.991 ± 0.000	0.002 ± 0.000
Carvalhal mandarin	0	0.003 ± 0.000	0.003 ± 0.000	0.815 ± 0.001	0.178 ± 0.001
Ladu mandarin	0	0.002 ± 0.000	0.002 ± 0.000	0.991 ± 0.000	0.003 ± 0.000
Se Hui Gan mandarin	0	0.002 ± 0.000	0.003 ± 0.000	0.991 ± 0.000	0.003 ± 0.000
Szibat mandarin	0	0.003 ± 0.000	0.002 ± 0.000	0.991 ± 0.000	0.003 ± 0.000
San Hu Hong Chu mandarin	0	0.003 ± 0.000	0.002 ± 0.000	0.991 ± 0.000	0.003 ± 0.000
Chandler pummelo	0	0.003 ± 0.000	0.002 ± 0.000	0.003 ± 0.000	0.991 ± 0.000
Da Xanh pummelo	0	0.003 ± 0.000	0.004 ± 0.000	0.008 ± 0.000	0.983 ± 0.000
Deep Red pummelo	0	0.003 ± 0.000	0.003 ± 0.000	0.003 ± 0.000	0.991 ± 0.000
Kao Pan pummelo	30	0.003 ± 0.000	0.003 ± 0.000	0.003 ± 0.000	0.989 ± 0.000
Nam Roi pummelo	4	0.003 ± 0.000	0.003 ± 0.000	0.004 ± 0.000	0.989 ± 0.000
Pink pummelo	3	0.003 ± 0.000	0.003 ± 0.000	0.003 ± 0.000	0.991 ± 0.000
Tahiti pummelo	1	0.003 ± 0.000	0.003 ± 0.000	0.003 ± 0.000	0.991 ± 0.000
Timorese pummelo	0	0.003 ± 0.000	0.003 ± 0.000	0.003 ± 0.000	0.991 ± 0.000
Corsican citron	0	0.002 ± 0.000	0.991 ± 0.000	0.002 ± 0.000	0.003 ± 0.000
Buddha's hand citron	0	0.003 ± 0.000	0.991 ± 0.000	0.002 ± 0.000	0.003 ± 0.000
Etrog citron	0	0.003 ± 0.000	0.991 ± 0.000	0.002 ± 0.000	0.003 ± 0.000
Humpang citron	0	0.003 ± 0.000	0.990 ± 0.000	0.003 ± 0.000	0.003 ± 0.000
Poncire citron	2	0.003 ± 0.000	0.991 ± 0.000	0.002 ± 0.000	0.003 ± 0.000
Mac Veu de montagne citron	2	0.003 ± 0.000	0.991 ± 0.000	0.002 ± 0.000	0.003 ± 0.000
Citrus micrantha	0	0.991 ± 0.000	0.003 ± 0.000	0.002 ± 0.000	0.003 ± 0.000
Citrus micrantha	0	0.991 ± 0.000	0.003 ± 0.000	0.002 ± 0.000	0.003 ± 0.000
Combava	0	0.722 ± 0.003	0.003 ± 0.000	0.032 ± 0.003	0.241 ± 0.002
Alemow	0	0.486 ± 0.002	0.505 ± 0.002	0.003 ± 0.000	0.004 ± 0.000
Excelsa lime	0	0.440 ± 0.003	0.549 ± 0.003	0.004 ± 0.000	0.006 ± 0.000
Mexican lime	0	0.503 ± 0.002	0.490 ± 0.002	0.003 ± 0.000	0.003 ± 0.000
Sweet Palestinian lime	0	0.003 ± 0.000	0.468 ± 0.003	0.319 ± 0.004	0.208 ± 0.005
Eureka lemon	0	0.013 ± 0.001	0.502 ± 0.006	0.334 ± 0.005	0.149 ± 0.005
Lisbon lemon	4	0.014 ± 0.001	0.520 ± 0.007	0.325 ± 0.007	0.140 ± 0.003
Marrakech limonette	0	0.012 ± 0.001	0.496 ± 0.005	0.337 ± 0.004	0.152 ± 0.004
Meyer lemon	0	0.003 ± 0.000	0.506 ± 0.006	0.326 ± 0.006	0.163 ± 0.003
Rangpur lime	0	0.003 ± 0.000	0.495 ± 0.002	0.496 ± 0.002	0.004 ± 0.000
Volckamer lemon	0	0.003 ± 0.000	0.475 ± 0.002	0.516 ± 0.002	0.005 ± 0.000
Tarocco sweet orange	0	0.003 ± 0.000	0.003 ± 0.000	0.600 ± 0.003	0.393 ± 0.003
Valencia late sweet orange	0	0.003 ± 0.000	0.003 ± 0.000	0.602 ± 0.002	0.390 ± 0.002

Washington navel sweet orange	0	0.003 ± 0.000	0.003 ± 0.000	0.602 ± 0.002	0.391 ± 0.001
Bouquet de fleurs sour orange	1	0.003 ± 0.000	0.003 ± 0.000	0.485 ± 0.002	0.507 ± 0.002
Citrus myrtifolia	2	0.003 ± 0.000	0.003 ± 0.000	0.483 ± 0.001	0.510 ± 0.001
Sevillan sour orange	0	0.003 ± 0.000	0.003 ± 0.000	0.483 ± 0.003	0.509 ± 0.003
Duncan grapefruit	0	0.003 ± 0.000	0.003 ± 0.000	0.378 ± 0.002	0.615 ± 0.002
Marsh grapefruit	1	0.003 ± 0.000	0.003 ± 0.000	0.368 ± 0.002	0.624 ± 0.002
Star Ruby grapefruit	1	0.003 ± 0.000	0.003 ± 0.000	0.364 ± 0.001	0.629 ± 0.001
Fallglo mandarin	0	0.003 ± 0.000	0.002 ± 0.000	0.713 ± 0.001	0.280 ± 0.001
Fortune mandarin	0	0.003 ± 0.000	0.004 ± 0.000	0.740 ± 0.001	0.251 ± 0.001
Fremont mandarin	0	0.003 ± 0.000	0.002 ± 0.000	0.746 ± 0.001	0.246 ± 0.001
Wilking mandarin	0	0.004 ± 0.000	0.003 ± 0.000	0.893 ± 0.000	0.097 ± 0.000
Clemenules clementine	0	0.003 ± 0.000	0.002 ± 0.000	0.758 ± 0.003	0.235 ± 0.003
Fina clementine	0	0.003 ± 0.000	0.003 ± 0.000	0.759 ± 0.001	0.233 ± 0.001
Afourer tangor	0	0.003 ± 0.000	0.002 ± 0.000	0.869 ± 0.001	0.124 ± 0.001
Ellendale tangor	0	0.007 ± 0.000	0.003 ± 0.000	0.890 ± 0.001	0.098 ± 0.001
Kiyomi tangor	1	0.003 ± 0.000	0.003 ± 0.000	0.631 ± 0.002	0.362 ± 0.002
Murcott tangor	0	0.003 ± 0.000	0.002 ± 0.000	0.844 ± 0.001	0.149 ± 0.001
Ortanique tangor	0	0.003 ± 0.000	0.003 ± 0.000	0.713 ± 0.001	0.28 ± 0.001
Temple Tangor	0	0.003 ± 0.000	0.003 ± 0.000	0.766 ± 0.002	0.227 ± 0.002
Minneola tangelo	0	0.003 ± 0.000	0.003 ± 0.000	0.710 ± 0.002	0.282 ± 0.002
Nova tangelo	0	0.003 ± 0.000	0.002 ± 0.000	0.688 ± 0.002	0.305 ± 0.002
Orlando tangelo	0	0.003 ± 0.000	0.003 ± 0.000	0.714 ± 0.001	0.279 ± 0.002
Triumph orangelo	30	0.003 ± 0.000	0.003 ± 0.000	0.322 ± 0.003	0.670 ± 0.003
Citrus amblycarpa	1	0.478 ± 0.002	0.005 ± 0.000	0.511 ± 0.003	0.005 ± 0.000

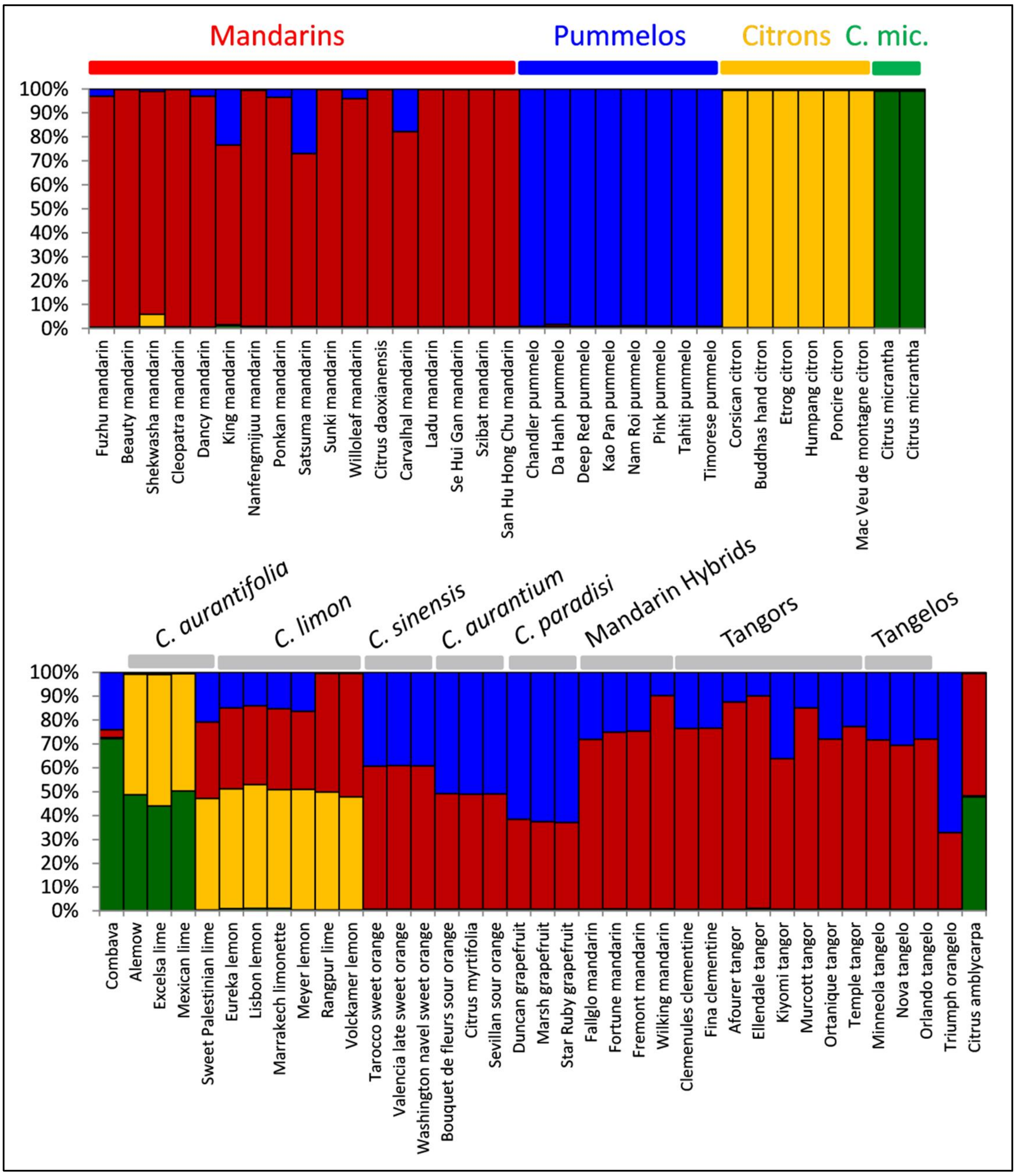

Figure A: Structure analysis of 70 Citrus cultivars from genotyping data with 103 SNP markers. Red, blue, yellow, and green correspond to the inferred contributions of C. reticulata, C. maxima, C. medica, and C. micrantha, respectively
